# Fragilides U–W: New 11,20-Epoxybriaranes from the Sea Whip Gorgonian Coral *Junceella fragilis*

**DOI:** 10.3390/md17120706

**Published:** 2019-12-15

**Authors:** Tung-Pin Su, Chien-Han Yuan, Yi-Ming Jhu, Bo-Rong Peng, Zhi-Hong Wen, Yu-Jen Wu, Tung-Ying Wu, Hong-Wen Liu, Ping-Jyun Sung

**Affiliations:** 1 Graduate Institute of Marine Biology, National Dong Hwa University, Pingtung 944, Taiwan; g3xz84120@yahoo.com.tw; 2 National Museum of Marine Biology and Aquarium, Pingtung 944, Taiwan; pengpojung@gmail.com; 3 Department of Otolaryngology, Kaohsiung Armed General Hospital, Kaohsiung 802, Taiwan; han86449@gmail.com (C.-H.Y.); edwinchu77@gmail.com (Y.-M.J.); 4 Department of Marine Biotechnology and Resources, National Sun Yat-sen University, Kaohsiung 804, Taiwan; wzh@mail.nsysu.edu.tw; 5 Department of Nursing, Meiho University, Pingtung 912, Taiwan; x00002180@meiho.edu.tw; 6 Department of Biological Science & Technology, Meiho University, Pingtung 912, Taiwan; 7 Department of Food Science and Nutrition, Meiho University, Pingtung 912, Taiwan; 8 Antai Medical Care Corporation Antai Tian-Sheng Memorial Hospital, Pingtung 928, Taiwan; 9 Chinese Medicine Research and Development Center, China Medical University Hospital, Taichung 404, Taiwan; 10 Graduate Institute of Natural Products, Kaohsiung Medical University, Kaohsiung 807, Taiwan

**Keywords:** *Junceella fragilis*, fragilide, briarane, gorgonian, junceellonoid, junceellin, iNOS

## Abstract

Three new 11,20-epoxybriaranes—fragilides U–W (**1**–**3**), as well as two known metabolites, junceellonoid D (**4**) and junceellin (**5**), were obtained from the octocoral *Junceella fragilis*. The structures of briaranes **1**–**3** were elucidated by spectroscopic methods and briaranes **3** and **5** displayed inhibition effects on inducible nitric oxide synthase (iNOS) release from RAW264.7.

## 1. Introduction

**Figure 1:**
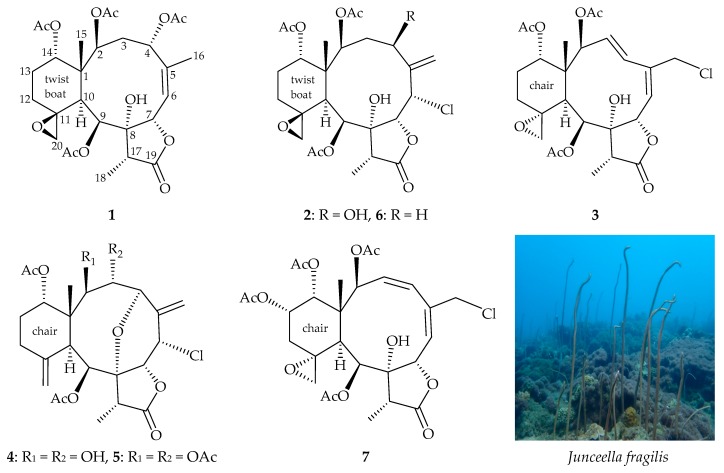
Structures of fragilides U–W (**1**–**3**), junceellonoid D (**4**), junceellin (**5**), robustolide F (**6**), juncenolide M (= frajunolide S) (**7**), and a picture of *Junceella fragilis*.

Gorgonian corals belonging to the genus *Junceella* (family Ellisellidae) [1,2,3], distributed abundantly in the coral reefs of tropical Indo-Pacific Ocean have been found to produce briarane diterpenoids, natural products of a marine origin, in abundance [4]. In our further research into the natural products of *Junceella fragilis* (Ridley 1884) (Figure 1), which was distributed extensively in the waters of Southern Taiwan, have resulted in the isolation of three new 11,20-epoxybriaranes– fragilides U–W (**1**–**3**) along with two know compounds junceellonoid D (**4**) [5] and junceellin (**5**) [6,7,8,9,10,11,12,13,14,15] (Figure 1). An anti-inflammatory assay was employed to evaluate the activity of these isolates against the release of inducible nitric oxide synthase (iNOS) from macrophage cells RAW264.7.

## 2. Results and Discussion

Fragilide U (**1**) was isolated as an amorphous powder and displayed a sodiated pseudomolecular ion at *m/z* 589.22583 in the (+)-HRESIMS, which suggested that the molecular formula of **1** was C_28_H_38_O_12_ (calcd. for C_28_H_38_O_12_ + Na, 589.22555) (Ω = 10). The IR spectrum of **1** showed the presence of hydorxy (ν_max_ 3445 cm^−1^), γ-lactone (ν_max_ 1780 cm^−1^), and ester (ν_max_ 1733 cm^−1^) groups. Analysis of the ^1^H, ^13^C NMR, and distortionless enhancement by polarization transfer (DEPT) spectra together with the molecular formula, suggested that there must be an exchangeable proton. The ^13^C NMR spectrum (Table 1), in combination with DEPT and heteronuclear single quantum coherence (HSQC) spectra, revealed the presence of four acetoxy groups (δ_C_ 21.7, 21.2, 20.9, 20.8, 4 × CH_3_; δ_C_ 170.1, 169.8, 169.4, 169.0, 4 × C), a γ-lactone moiety (δ_C_ 175.9), and a trisubstituted olefin (δ_C_ 142.0, C; 120.6, CH). Base on the ^13^C NMR data and numbers of unsaturation, **1** was established as a diterpenoid featuring with four rings. An exocyclic epoxy group was deduced from the signals of an oxygenated quaternary carbon and an oxymethylene at δ_C_ 62.5 and 59.1, respectively, and further supported by the chemical shifts of oxymethylene protons at δ_H_ 2.85 (1H, d, *J* = 4.4 Hz) and 2.98 (1H, dd, *J* = 4.4, 1.6 Hz). Moreover, a methyl singlet (δ_H_ 1.09, 3H, s), a methyl doublet (δ_H_ 1.15, 3H, d, *J* = 7.6 Hz), a vinyl methyl (δ_H_ 2.00, 3H, d, *J* = 1.6 Hz), three pairs of aliphatic methylene protons (δ_H_ 2.04, 1H, m; 2.62, 1H, ddd, *J* = 18.4, 4.0, 2.4 Hz; 1.13, 1H, m; 2.30, 1H, m; 2.11, 1H, m; 1.81, 1H, m), two aliphatic methine protons (δ_H_ 2.36, 1H, dd, *J* = 5.6, 1.6 Hz; 2.33, 1H, q, *J* = 7.6 Hz), five oxymethine protons (δ_H_ 5.91, 1H, br s; 5.65, 1H, d, *J* = 5.6 Hz; 5.04, 1H, d, *J* = 8.8 Hz; 5.03, 1H, br s; 4.71, 1H, d, *J* = 4.8 Hz), an olefin proton (δ_H_ 5.67, 1H, dq, *J* = 8.8, 1.6 Hz), four acetate methyls (δ_H_ 2.24, 2.01, 1.99, 1.98, each 3H × s), and a hydroxy proton (δ_H_ 4.85, 1H, br s) were observed in the ^1^H NMR spectrum (Table 2).

**Figure 2:**
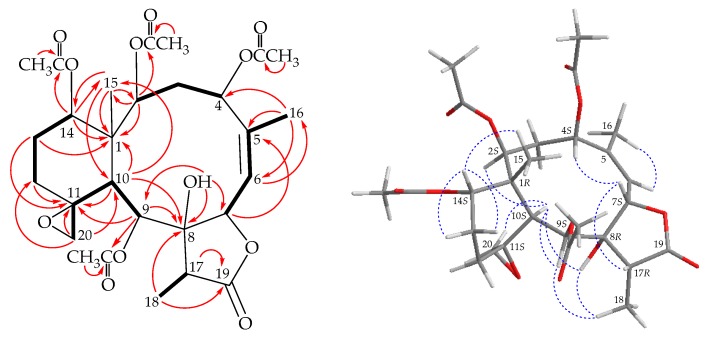
The correlation spectroscopy (COSY) (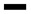) correlations, selective heteronuclear multiple bond correlation (HMBC) experiment (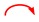), and protons with key nuclear Overhauser effect spectroscopy (NOESY) (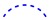) correlations of **1**.

Coupling information in the correlation spectroscopy (COSY) analysis enabled the proton sequences from H-2/H_2_-3/H-4, H-6/H-7, H-9/H-10, H_2_-12/H_2_-13/H-14, and H-17/H_3_-18 (Figure 2), which was assembled with a heteronuclear multiple bond correlation (HMBC) experiment (Figure 2). The HMBC between protons and quaternary carbons, such as H-2, H-10, H-13β, H-14, H_3_-15/C-1; H-7, H_3_-16/C-5; H-9, H-10, H_3_-18/C-8; H-9, H-10, H_2_-12, H-13α, H_2_-20/C-11; and H-17, H_3_-18/C-19, permitted elucidation of the carbon skeleton of **1**. A vinyl methyl at C-5 was confirmed by the HMBC between H_3_-16/C-4, C-5, C-6 and H-6/C-16; and further supporting by an allylic coupling between H-6 and H_3_-16 (*J* = 1.6 Hz). The methyl group on C-1 (Me-15) was substantiated by the HMBC from H_3_-15/C-1, C-2, C-10, C-14; and H-2, H-10, H-14/C-15. The epoxy group at C-11/20 was confirmed by the HMBC between H_2_-20/C-10, C-11; and H-20a/C-12; and further supporting by a long range ^4^*J*-^1^H–^1^H correlation between H-10 (δ_H_ 2.36) and H-20b (δ_H_ 2.98) (*J* = 1.6 Hz). A hydroxy group attaching at C-8 was to infer that an HMBC of a hydroxy proton at δ_H_ 4.85 to C-7, C-8, and C-9. Moreover, HMBC from the oxymethine protons at δ_H_ 5.03 (H-2), 5.65 (H-9), and 4.71 (H-14) to the acetate carbonyls at δ_C_ 169.0, 169.4, and 170.1, placed the acetoxy groups on C-2, C-9, and C-14, respectively. Ten of the 12 oxygen atoms in the molecular formula of **1** could be accounted for the presence of a γ-lactone, three esters, an epoxide, and a hydroxy group. Thus, the remaining two oxygen atoms had to be positioned at C-4 as an acetoxy group, as indicated by its ^1^H and ^13^C NMR chemical shifts (δ_H_ 5.91, 1H, br s; δ_C_ 70.6, CH), although no HMBC was observed from H-4 to any acetate carbonyl.

Based on a summary of the ^13^C chemical shifts of 11,20-epoxy group in naturally occurring briarane analogues, with ^13^C NMR data for C-11 and C-20 at δ_C_ 62–63 and 58–60 ppm, the epoxide group was β-oriented and the cyclohexane ring existed in a twist boat conformation [16]; thus, the configuration of the 11,20-epoxy group in **1** should be β-oriented and the cyclohexane ring was found to be in a twist boat conformation for the ^13^C chemical shifts at δ_C_ 62.5 (C-11) and 59.1 (CH_2_- 20). The relative stereochemistry of **1** was established by the analysis of correlations observed in a nuclear Overhauser effect spectroscopy (NOESY) experiment (Figure 2). In the NOESY spectrum of **1**, NOE correlations between H-10/H-2, H-10/H-9, and H-10/OH-8, while no correlation was seen between H-10 and H_3_-15, suggesting that these protons H-2, H-9, and H-10, and the hydroxy group at C-8 were α-oriented; meanwhile, an NOE correlation of H_3_-15 with H-14 indicated that H-14 was β-oriented. The NOESY spectrum showed a correlation from H-6 to H_3_-16, revealing the *Z* geometry of C-5/6 double bond. H_3_-18 exhibited NOE correlations to OH-8 and H-9, suggesting the α-orientation of Me-18 at C-17. H-7 displayed NOE correlations with H-4 and H-17, which further confirmed that these three protons were in β-orientation at C-7, C-4, and C-17. Based on the above findings, the relative configurations of stereogenic carbons of **1** were elucidated as 1R*,2S*,4S*,7S*, 8R*,9S*,10S*,11S*,14S* and 17R*. However, as briaranes **1**–**4** were isolated along with the known chlorinated briarane, junceellin (**5**) [6], and the structure, including the absolute configuration of junceellin (**5**) was further confirmed by a single-crystal X-ray diffraction analysis [7,15]. It is reasonable, therefore, on biogenetic grounds to assume that briaranes **1**–**4** have the same absolute configuration as that of **5**. Therefore, the configurations of the stereogenic carbons of **1** should be elucidated as 1*R*,2*S*,4*S*,7*S*,8*R*,9*S*,10*S*,11*S*,14*S* and 17*R* (Supplementary Materials, Figures S1–S8).

**Figure 3:**
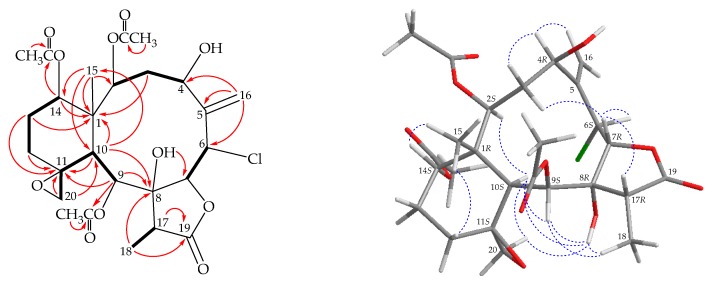
The COSY (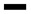) correlations, selective HMBC (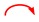), and protons with key NOESY (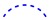) correlations of **2**.

Our present study has also led to the isolation of a new briarane, fragilide V (**2**). The molecular formula of C_26_H_35_ClO_11_ was deduced from (+)-HRESIMS at *m/z* 581.17589 (calcd. for C_26_H_35_^35^ClO_11_ + Na, 581.17601). Carbonyl resonances in the ^13^C NMR spectrum of **2** (Table 1) at δ_C_ 175.2, 171.0, 170.1, and 169.7 revealed the presence of a γ-lactone and three esters. In the ^1^H NMR spectrum of **2** (Table 2), the signals for three acetate methyls were observed at δ_H_ 2.23, 2.03, and 1.99. It was found that the 1D (Table 1 and Table 2) and 2D NMR (Figure 3) data of **2** were similar to those of a known briarane, robustolide F (**6**) [17,18] (Figure 1), except that the signals corresponding to a hydroxy group in **2** were replaced by signals for a proton in **6**. In the NOESY spectrum, one of the C-3 methylene protons (δ_H_ 2.38) showed a correlation to H-7 and not with H-2, suggesting the β-orientation of this proton by modeling study and the other was assigned as H-3α (δ_H_ 1.75). A correlation from H-4 to H-3α, suggested that H-4 was α-oriented according to modeling analysis. Therefore, the configuration of the stereogenic carbons of **2** were elucidated as 1*R*,2*S*,4*R*,6*S*,7*R*,8*R*,9*S*,10*S*,11*S*,14*S,* and 17*R* (Figure 3) (Supplementary Materials, Figures S9–S16).

**Figure 4:**
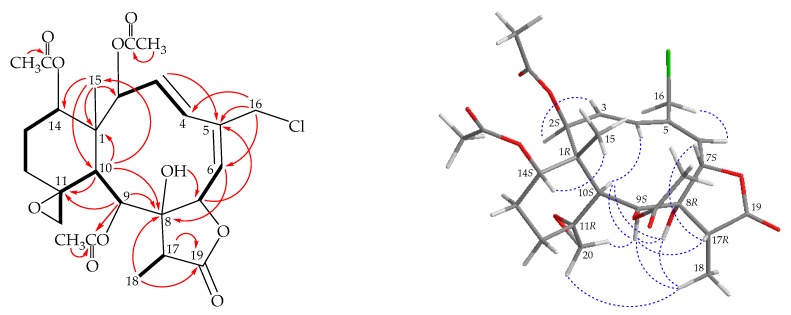
The COSY (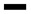) correlations, selective HMBC (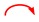), and protons with key NOESY (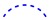) correlations of **3**.

Briarane **3** (fragilide W) was found to have a molecular formula of C_26_H_33_ClO_10_ based on its (+)- HRESIMS at *m/z* 563.16554 (calcd. for C_26_H_33_^35^ClO_10_ + Na, 563.16545). Its absorption peaks in the IR spectrum showed ester, γ-lactone, and broad OH stretching at 1738, 1777, and 3459 cm^−1^, respectively. The ^13^C NMR spectrum indicated that three esters and a γ-lactone were present, as carbonyl resonances were observed at δ_C_ 174.8, 170.4, 169.7, 169.3 (Table 1). The ^1^H NMR spectrum indicated the presence of three acetate methyls (δ_H_ 2.27, 2.25, 2.11, each 3H × s) (Table 2). The ^1^H and ^13^C NMR spectra of **3** was found to be similar with those of a known briarane, juncenolide M (= frajunolide S) (**7**) (Figure 1), isolated from *J. juncea* and *J. fragilis* [19,20], except that the signals corresponding to the 13-acetoxy and 3(*Z*)-ene moieties in **7** were disappeared and replaced by a proton and an (*E*)-ene moieties in **3**, respectively. The locations of the functional groups were confirmed by 2D-NMR correlations (Figure 4), and hence the structure of fragilide W was assigned as **3**, and the configurations of the stereogenic carbons were elucidated as 1*R*,2*S*,7*S*,8*R*,9*S*,10*S*,11*R*, 14*S,* and 17*R* (Figure 4) (Supplementary Materials, Figures S17–S24).

The known compound **4** was found to be identical with the known junceellonoid D, on the basis of the comparison of its physical and spectroscopic data with those of reported previously [5,21] (Supplementary Materials, Figures S25–S32).

**Figure 5:**
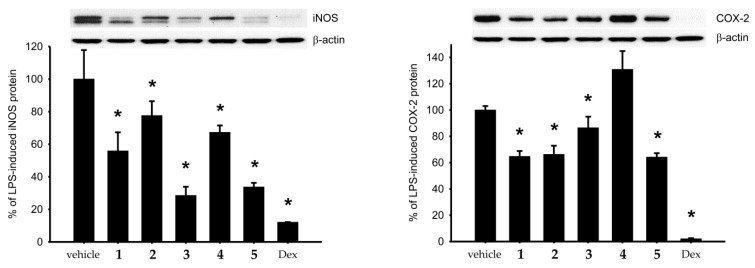
Activities of briaranes **1**–**5** on the expression of inducible nitric oxide synthase (iNOS) and cyclooxygenase-2 (COX-2) proteins in LPS-treated murine RAW264.7 macrophage cells. Western blotting showed that briaranes **3** and **5** reduced the expression of iNOS. Data were normalized to the cells treated with LPS only, and cells treated with dexamethasone were used as a positive control. Data are expressed as the mean ± SEM (*n* = 3). * Significantly different from cells treated with LPS (*p* < 0.05).

Using an *in vitro* pro-inflammatory suppression assay, the activities of briaranes **1**–**5** on the release of iNOS and cyclooxygenase-2 (COX-2) protein from lipopolysaccharides (LPS)-stimulated RAW264.7 were assayed (Figure 5 and Table 3). The results showed that briaranes **3** and **5** reduced the release of iNOS to 28.55 and 33.72% at a concentration of 10 μM. Briarane **4** was found to be more weak in terms of reducing the expression of iNOS, indicating that the activity of briaranes **4** and **5** is largely dependent on the functional groups at C-2 and C-3. It is interesting to note that briarane **4** was found to enhance the expression of COX-2 to 130.88%, at a concentration of 10 μM.

## 3. Materials and Methods

### 3.1. General Experimental Procedures

Melting points were determined using a Fargo apparatus and the values were uncorrected. 1D and 2D NMR spectra were recorded on a 600 MHz Jeol NMR (model ECZ600R, Tokyo, Japan) or on a 400 MHz Jeol NMR (model ECZ400S) spectrometers using the residual CHCl_3_ signal (δ_H_ 7.26 ppm) and CDCl_3_ (δ_C_ 77.1 ppm) as internal standards for ^1^H and ^13^C NMR, respectively. ESIMS and HRESIMS were obtained from the Bruker mass spectrometer with 7 Tesla magnets (model: SolariX FTMS system, Bremen, Germany). Column chromatography, HPLC, IR, and optical rotation were performed according to our earlier research [15]. 

### 3.2. Animal Material

Specimens of *J. fragilis* used for this study were collected in April 2017 by self-contained underwater breathing apparatus (SCUBA) at depths of 10−15 m off the coast of South Bay, Kenting, Taiwan. The samples were stored in a −20 °C freezer until extraction. A voucher specimen was deposited in the NMMBA (voucher no.: NMMBA-TW-GC-2017-022). Identification of the species of this organism was performed by comparison as described in previous studies [1,2,3].

### 3.3. Extraction and Isolation

Sliced bodies (wet/dry weight = 795/313 g) of the coral specimen were prepared and extracted with a 1:1 mixture of methanol (MeOH) and dichloromethane (CH_2_Cl_2_) (1:1) to give a crude extract (19.0 g) which was partitioned between ethyl acetate (EtOAc) and H_2_O. The EtOAc extract (8.0 g) was then applied to a silica gel column chromatograph (C.C.) and eluted with gradients of *n*- hexane/acetone (stepwise from 50:1 to 1:2; volume ratio) to furnish 8 fractions (fractions: A−H). Fraction F was chromatographed on silica gel C.C. and eluted with gradients of *n*-hexane/EtOAc (2:1 to 1:2, stepwise) to furnish 4 fractions (fractions F1−F4). Fraction F3 was washed with a mixture of *n*-hexane/acetone (30:1) and the undissolved **5** (23.5 mg) was obtained. Fraction G was purified by normal-phase HPLC (NP-HPLC) using a mixture of *n*-hexane and EtOAc (4:1 to 1:1, stepwise) to afford 16 fractions (fractions G1−G16). Afterward, fraction G15 was separated by NP-HPLC using a mixture of CH_2_Cl_2_ and acetone (v:v = 10:1; at a flow rate = 2.0 mL/min) to yield **1** (1.7 mg). Fraction G14 was separated by NP-HPLC using a mixture of *n*-hexane/acetone (2:1; volume ratio) to yield 6 fractions (fractions: G14A−G14F). Fractions G14C and G14D were combined and purified by reverse-phase HPLC (RP-HPLC) using a mixture of MeOH and H_2_O (v:v = 65:35; at a flow rate = 4.0 mL/min) to afford **2** (0.8 mg). Fraction G9 was purified by RP-HPLC using a mixture of MeOH and H_2_O (v:v = 60:40; at a flow rate = 4.0 mL/min) to yield 16 fractions (fractions G9A−G9P), including compound **3** (0.8 mg, G9D). Fraction G9M was separated by RP-HPLC using a mixture of acetonitrile and H_2_O (v:v = 55:45; at a flow rate = 4.0 mL/min) to obtain **4** (0.8 mg). 

Fragilide U (**1**): Amorphous powder; ${\left[\alpha \right]}_{D}^{24}$ −12 (*c* 0.09, CHCl_3_); IR (KBr) ν_max_ 3445, 1780, 1733 cm^−1^; ^13^C (100 MHz, CDCl_3_) and ^1^H (400 MHz, CDCl_3_) NMR data, see Table 1; Table 2; ESIMS: *m/z* 589 [M + Na]^+^; HRESIMS: *m/z* 589.22583 (calcd. for C_28_H_38_O_12_ + Na, 589.22555).

Fragilide V (**2**): Amorphous powder; ${\left[\alpha \right]}_{D}^{23}$ −26 (*c* 0.04, CHCl_3_); IR (KBr) ν_max_ 3444, 1779, 1738 cm^−1^; ^13^C (100 MHz, CDCl_3_) and ^1^H (400 MHz, CDCl_3_) NMR data, see Table 1; Table 2; ESIMS: *m/z* 581 [M + Na]^+^, 583 [M + 2 + Na]^+^; HRESIMS: *m/z* 581.17589 (calcd. for C_26_H_35_^35^ClO_11_ + Na, 581.17601).

Fragilide W (**3**): Amorphous powder; ${\left[\alpha \right]}_{D}^{23}$ −21 (*c* 0.04, CHCl_3_); IR (KBr) ν_max_ 3459, 1777, 1738 cm^−1^; ^13^C (150 MHz, CDCl_3_) and ^1^H (600 MHz, CDCl_3_) NMR data, see Table 1; Table 2; ESIMS: *m/z* 563 [M + Na]^+^, 565 [M + 2 + Na]^+^; HRESIMS: *m/z* 563.16554 (calcd. for C_26_H_33_^35^ClO_10_ + Na, 563.16545).

Junceellonoid D (**4**): Amorphous powder; ${\left[\alpha \right]}_{D}^{24}$ −18 (*c* 0.04, CHCl_3_) (ref. [5] ${\left[\alpha \right]}_{D}^{\;}$ −44.8 (*c* 0.10, CHCl_3_, MeOH); ref. [21] ${\left[\alpha \right]}_{D}^{23}$ −31 (*c* 0.05, CHCl_3_)); IR (ATR) ν_max_ 3509, 1793, 1733 cm^−1^; ^13^C and ^1^H NMR data were found to be in full agreement with those reported previously [21]; ESIMS: *m/z* 521 [M + Na]^+^, 523 [M + 2 + Na]^+^; HRESIMS: *m/z* 521.15469 (calcd. for C_24_H_31_^35^ClO_9_ + Na, 521.15488).

Junceellin (**5**): Colorless crystals; mp 277–278 °C (ref. [15] mp 272–275 °C); ${\left[\alpha \right]}_{D}^{23}$ −3 (*c* 1.18, CHCl_3_) (ref. [15] ${\left[\alpha \right]}_{D}^{25}$ −2 (*c* 0.89, CHCl_3_)); IR (KBr) ν_max_ 1794, 1743 cm^−1^; ^13^C and ^1^H NMR data were found to be in full agreement with those reported previously [6,8,9,12]; ESIMS: *m/z* 605 [M + Na]^+^, 607 [M + 2 + Na]^+^; HRESIMS: *m/z* 605.17612 (calcd. for C_28_H_35_^35^ClO_11_ + Na, 605.17601).

### 3.4. In Vitro Anti-Inflammatory Assay

The anti-inflammatory assay was employed to evaluate the activities of briaranes **1**–**5** reduce the release of iNOS and COX-2 from macrophage cells as the literature reported [22,23,24,25].

## 4. Conclusions

*J. fragilis* was proven to be a rich source to produce a wide structural diversity of briarane-type diterpenoids that possess various biomedical properties, particularly in anti-inflammatory activity [26,27]. In our continued study on *J. fragilis*, three previously unreported 11,20-epoxybriaranes, fragilides U–W (**1**–**3**), along with two known briaranes, junceellonoid D (**4**) and junceellin (**5**), were isolated. The exocyclic 11,20-epoxy group was proven to be a chemical marker for briarane-type natural products from the gorgonian corals belonging to the family Ellisellidae [28]. In the present study, the anti-inflammatory activity of **1**–**5** was assayed using inhibition of iNOS and COX-2 and the results indicated that fragilide W (**3**) and junceellin (**5**) showed the most potent suppressive effect on iNOS release.

## Figures and Tables

**Table 1:** ^13^C NMR data for briaranes **1**–**3**.

Position	1 ^a^	2 ^a^	3 ^b^
1	47.0, C ^c^	46.1, C	49.1, C
2	72.2, CH	72.1, CH	75.4, CH
3	37.5, CH_2_	37.6, CH_2_	136.4, CH
4	70.6, CH	73.4, CH	128.2, CH
5	142.0, C	144.1, C	135.9, C
6	120.6, CH	58.0, CH	128.8, CH
7	76.5, CH	79.0, CH	80.0, CH
8	80.4, C	81.9, C	80.9, C
9	67.4, CH	68.9, CH	67.1, CH
10	39.9, CH	39.8, CH	39.5, CH
11	62.5, C	62.6, C	60.8, C
12	23.6, CH_2_	23.5, CH_2_	29.4, CH_2_
13	24.2, CH_2_	24.0, CH_2_	25.5, CH_2_
14	73.2, CH	73.5, CH	78.9, CH
15	14.6, CH_3_	15.4, CH_3_	16.0, CH_3_
16	21.4, CH_3_	121.0, CH_2_	48.0, CH_2_
17	42.4, CH	43.8, CH	45.2, CH
18	6.6, CH_3_	7.1, CH_3_	6.9, CH_3_
19	175.9, C	175.2, C	174.8, C
20	59.1, CH_2_	58.2, CH_2_	50.4, CH_2_
Acetate methyls	21.7, CH_3_	21.9, CH_3_	21.7, CH_3_
	21.2, CH_3_	21.0, CH_3_	21.4, CH_3_
	20.9, CH_3_	21.0, CH_3_	21.3, CH_3_
	20.8, CH_3_		
Acetate carbonyls	170.1, C	171.0, C	170.4, C
	169.8, C	170.1, C	169.7, C
	169.4, C	169.7, C	169.3, C
	169.0, C		

^a^ Spectra measured at 100 MHz in CDCl_3_. ^b^ Spectra measured at 150 MHz in CDCl_3_. ^c^ Multiplicity deduced by distortionless enhancement by polarization transfer (DEPT) and heteronuclear single quantum coherence (HSQC) spectra.

**Table 2:** ^1^H NMR data (*J* in Hz) for briaranes **1**–**3**.

Position	1 ^a^	2 ^a^	3 ^b^
2	5.03 br s	4.96 dd (2.8, 2.8)	5.63 d (6.0)
3α/β	2.04 m; 2.62 ddd (18.4, 4.0, 2.4)	1.75 m; 2.38 m	6.04 dd (17.4, 6.0)
4	5.91 br s	4.85 m	6.42 d (17.4)
6	5.67 dq (8.8, 1.6)	5.49 br s	5.66 d (3.6)
7	5.04 d (8.8)	5.15 d (2.4)	5.62 d (3.6)
9	5.65 d (5.6)	5.52 d (6.4)	4.77 d (6.6)
10	2.36 dd (5.6, 1.6)	2.31 d (6.4)	3.32 d (6.6)
12α/β	1.13 m; 2.30 m	1.15 m; 2.30 m	1.29 m; 2.18 m
13α/β	2.11 m; 1.81 m	2.14 m; 1.78 m	2.08 m; 1.85 dddd (12.0, 12.0, 3.6, 1.8)
14	4.71 d (4.8)	4.86 d (4.4)	4.94 dd (3.6, 3.0)
15	1.09 s	1.13 s	0.87 s
16a/b	2.00 d (1.6)	5.74 s; 5.97 s	4.13 d (12.0); 4.19 d (12.0)
17	2.33 q (7.6)	2.29 q (7.6)	2.36 q (7.2)
18	1.15 d (7.6)	1.22 d (7.6)	1.18 d (7.2)
20a/b	2.85 d (4.4); 2.98 dd (4.4, 1.6)	2.83 d (4.0); 2.85 br d (4.0)	2.76 d (2.4); 3.40 dd (2.4, 2.4)
OH-8	4.85 br s	4.63 br s	3.13 d (1.2)
Acetate methyls	2.24 s	2.23 s	2.27 s
	2.01 s	2.03 s	2.25 s
	1.99 s	1.99 s	2.11 s
	1.98 s		

^a^ Spectra measured at 400 MHz in CDCl_3_. ^b^ Spectra measured at 600 MHz in CDCl_3_.

**Table 3:** Effects of briaranes **1**–**5** on LPS-induced pro-inflammatory inducible nitric oxide synthase (iNOS) and cyclooxygenase-2 (COX-2) protein expression in macrophages at a concentration of 10 μM.

	iNOS	COX-2	β-Actin
Compound	Expression (% of LPS)		
Lipopolysaccharides	100.01 ± 17.81	100.00 ± 3.04	100.00 ± 6.05
1	55.88 ± 11.42	64.73 ± 4.07	90.05 ± 7.11
2	77.58 ± 8.82	66.23 ± 6.59	99.29 ± 5.24
3	28.55 ± 5.35	86.46 ± 8.46	101.69 ± 6.46
4	67.25 ± 4.27	130.88 ± 13.94	103.20 ± 2.56
5	33.72 ± 2.57	64.15 ± 3.03	84.52 ± 7.78
Dexamethasone	12.11 ± 0.03	2.11 ± 0.44	123.86 ± 2.99

Data were normalized to those of cells treated with LPS alone, and cells treated with dexamethasone were used as a positive control (10 μM). Data are expressed as the mean ± SEM (*n* = 3).

## Supplementary Materials

The Supplementary Materials are available online at https://www.mdpi.com/1660-3397/17/12/706/s1. HRESIMS, IR, 1D and 2D NMR spectra of compounds **1**–**4**.
