# Supplementary material for: Fragilides U–W: New 11,20-Epoxybriaranes from the Sea Whip Gorgonian Coral Junceella fragilis
Source: Mar Drugs. 2019 Dec 15;17(12):706. doi: 10.3390/md17120706 (PMC6950706; doi:10.3390/md17120706)
Supplement: Supplementary file 1 [file marinedrugs-17-00706-s001.pdf]

S1. HRESIMS spectrum of compound **1** 2
S2. IR spectrum of compound **1** 2
S3. ^{1}H NMR spectrum (400 MHz) of compound **1** in $CDCl_3$ 3
S4. ^{13}C NMR spectrum (100 MHz) of compound **1** in $CDCl_3$ 3
S5. HSQC spectrum of compound **1** in $CDCl_3$ 4
S6. HMBC spectrum of compound **1** in $CDCl_3$ 4
S7. ^{1}H-^{1}H COSY spectrum of compound **1** in $CDCl_3$ 5
S8. NOESY spectrum of compound **1** in $CDCl_3$ 5
S9. HRESIMS spectrum of compound **2** 6
S10. IR spectrum of compound **2** 6
S11. ^{1}H NMR spectrum (400 MHz) of compound **2** in $CDCl_3$ 7
S12. ^{13}C NMR spectrum (100 MHz) of compound **2** in $CDCl_3$ 7
S13. HSQC spectrum of compound **2** in $CDCl_3$ 8
S14. HMBC spectrum of compound **2** in $CDCl_3$ 8
S15. ^{1}H-^{1}H COSY spectrum of compound **2** in $CDCl_3$ 9
S16. NOESY spectrum of compound **2** in $CDCl_3$ 9
S17. HRESIMS spectrum of compound **3** 10
S18. IR spectrum of compound **3** 10
S19. ^{1}H NMR spectrum (600 MHz) of compound **3** in $CDCl_3$ 11
S20. ^{13}C NMR spectrum (150 MHz) of compound **3** in $CDCl_3$ 11
S21. HSQC spectrum of compound **3** in $CDCl_3$ 12
S22. HMBC spectrum of compound **3** in $CDCl_3$ 12
S23. ^{1}H-^{1}H COSY spectrum of compound **3** in $CDCl_3$ 13
S24. NOESY spectrum of compound **3** in $CDCl_3$ 13
S25. HRESIMS spectrum of compound **4** 14
S26. IR spectrum of compound **4** 14
S27. ^{1}H NMR spectrum (600 MHz) of compound **4** in $CDCl_3$ 15
S28. ^{13}C NMR spectrum (150 MHz) of compound **4** in $CDCl_3$ 15
S29. HSQC spectrum of compound **4** in $CDCl_3$ 16
S30. HMBC spectrum of compound **4** in $CDCl_3$ 16
S31. ^{1}H-^{1}H COSY spectrum of compound **4** in $CDCl_3$ 17
S32. NOESY spectrum of compound **4** in $CDCl_3$ 17

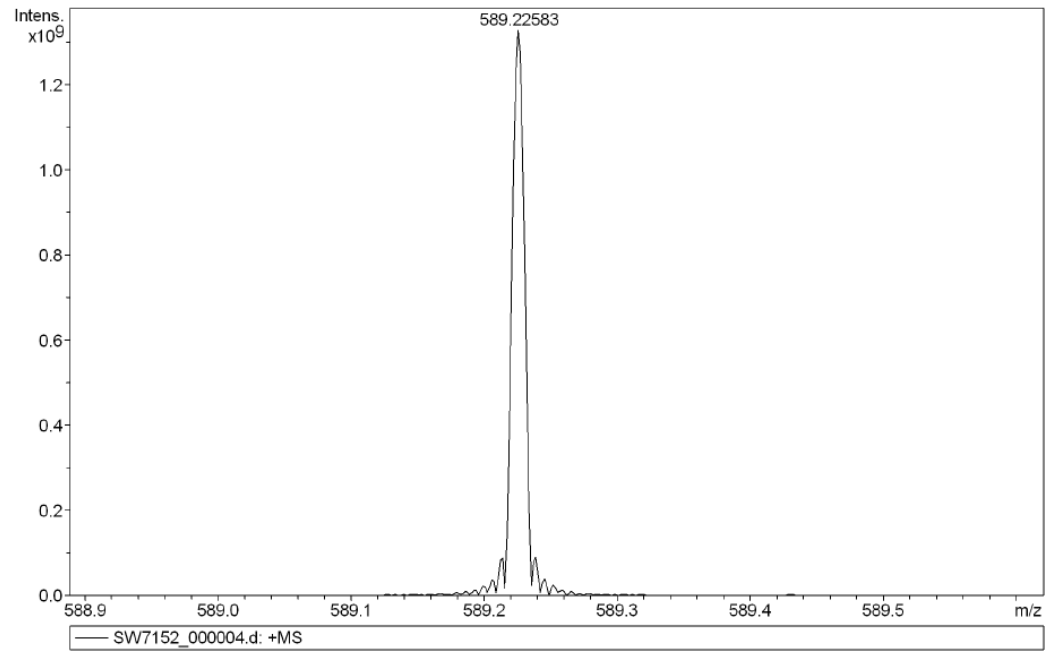

Meas. m/z	#	Formula	Score	m/z	err [mDa]	err [ppm]	mSigma	rdb	e⁻ Conf	N-Rule
589.22583	1	C 28 H 38 Na O 12	100.00	589.22555	-0.28	-0.48	21.7	9.5	even	ok

S1. HRESIMS spectrum of compound **1**

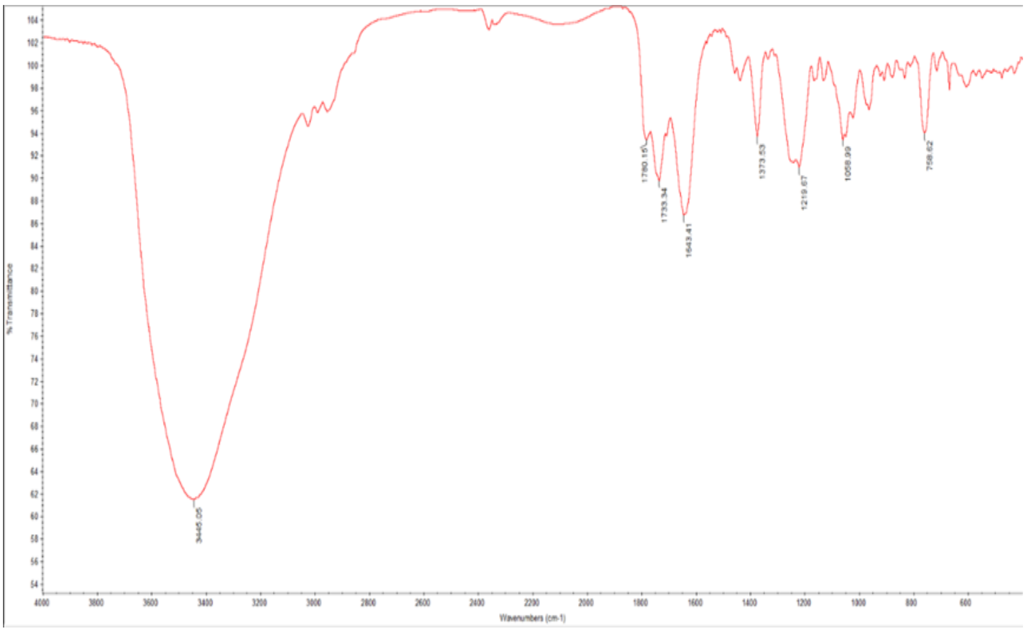

S2. IR spectrum of compound **1**

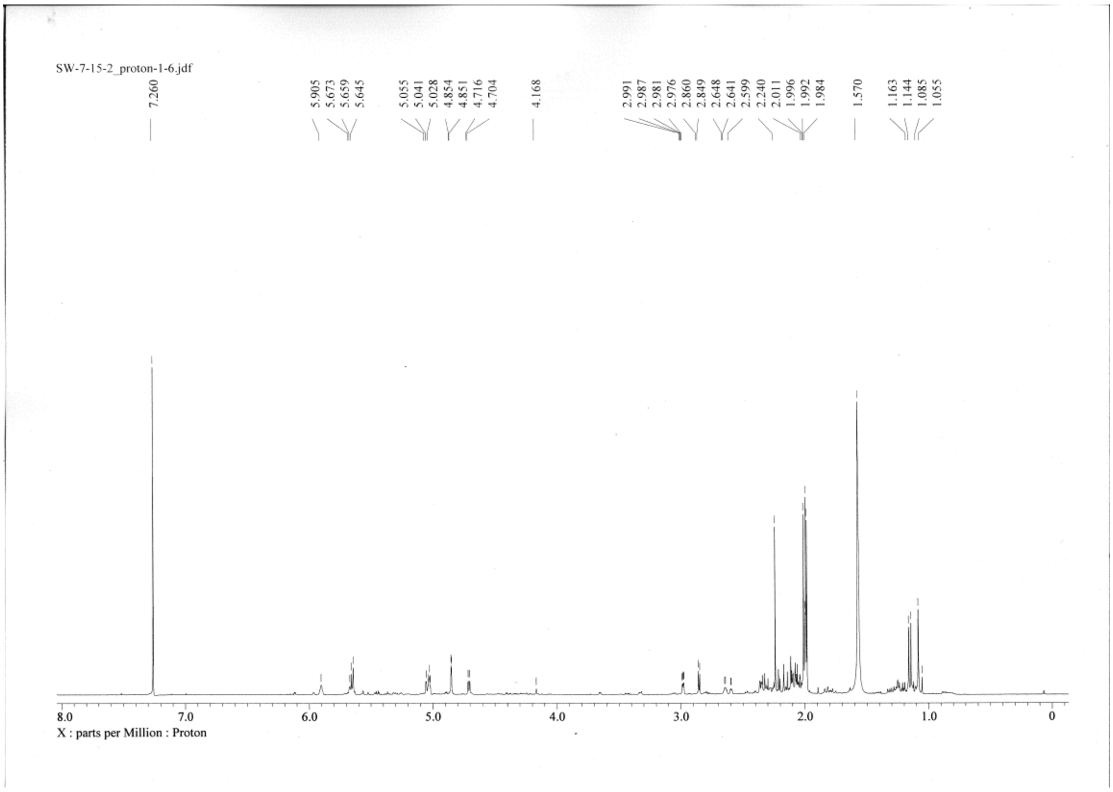

S3. ^{1}H NMR spectrum (400 MHz) of compound **1** in $CDCl_3$

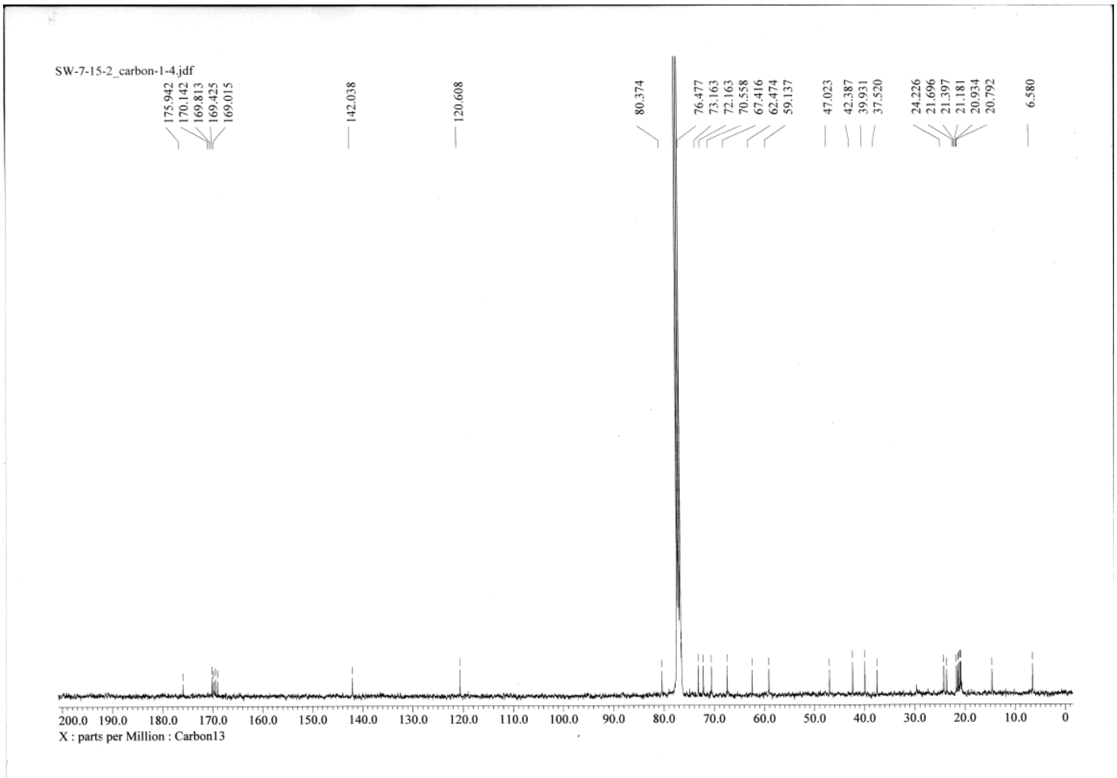

S4. ^{13}C NMR spectrum (100 MHz) of compound **1** in $CDCl_3$

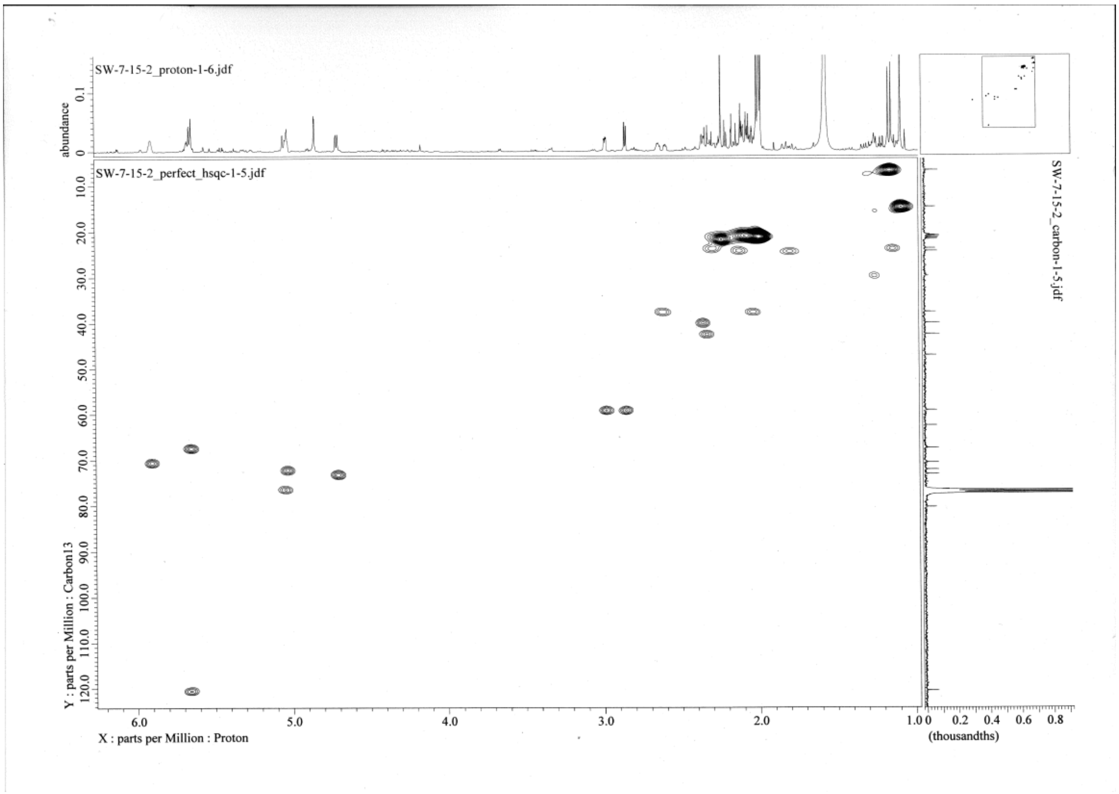

S5. HSQC spectrum of compound **1** in $CDCl_3$

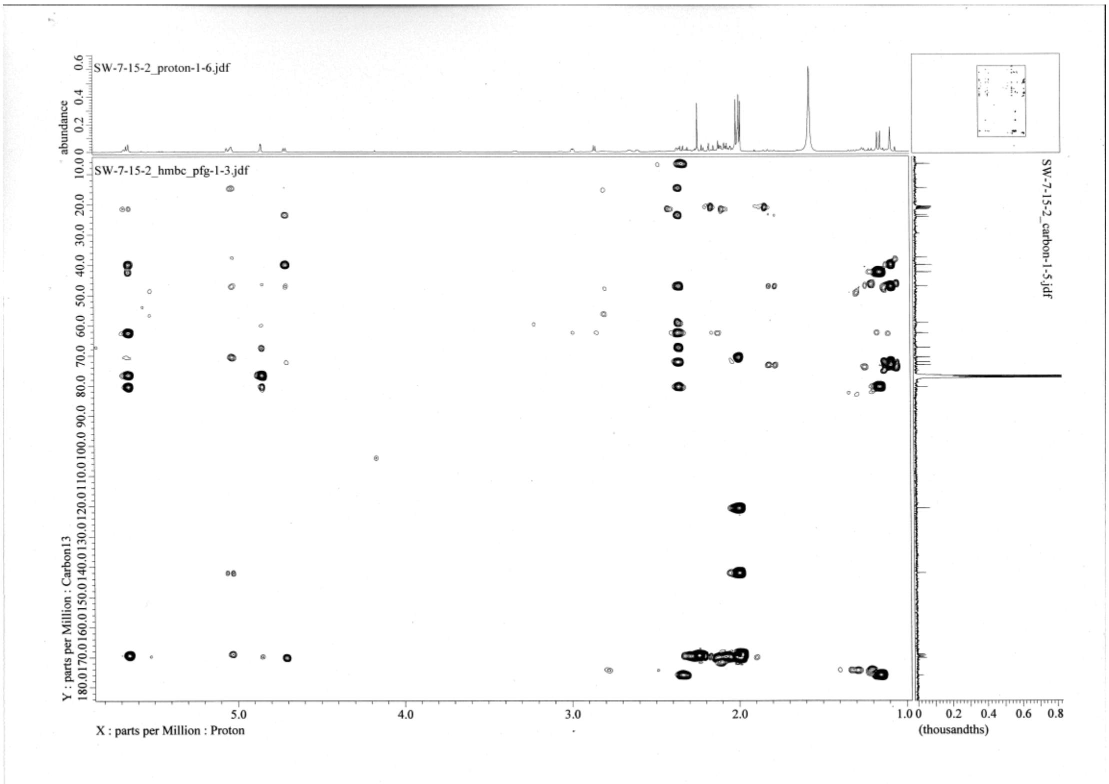

S6. HMBC spectrum of compound **1** in $CDCl_3$

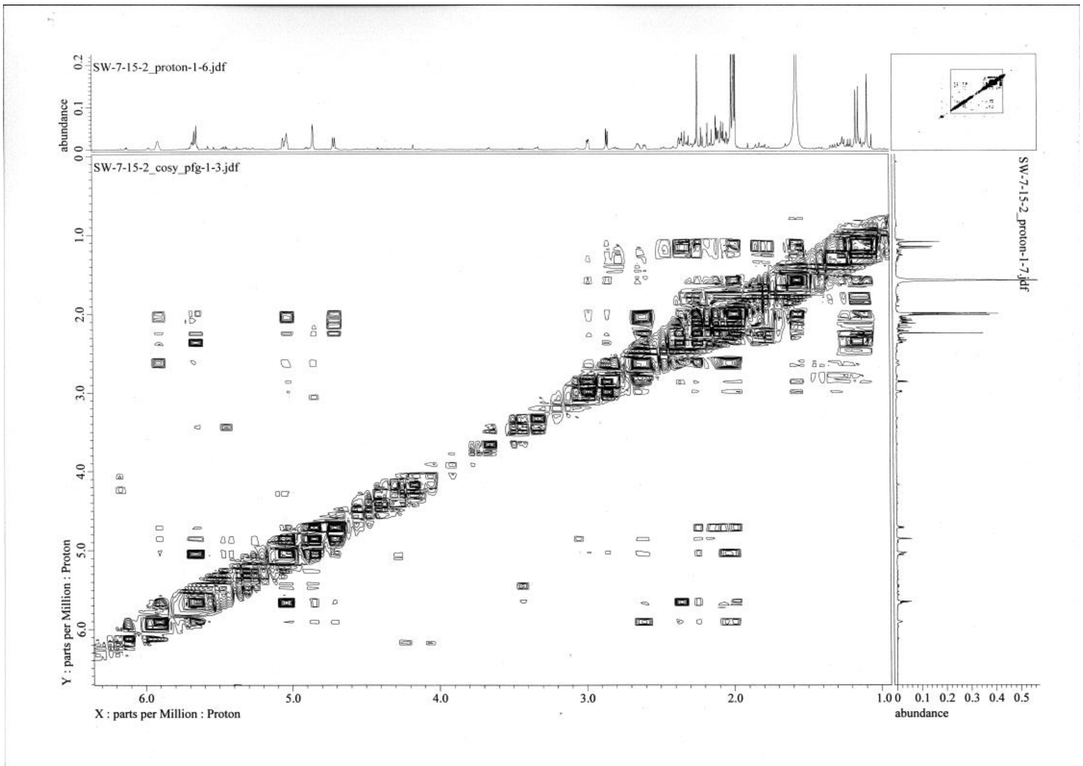

S7. ^{1}H-^{1}H COSY spectrum of compound **1** in $CDCl_3$

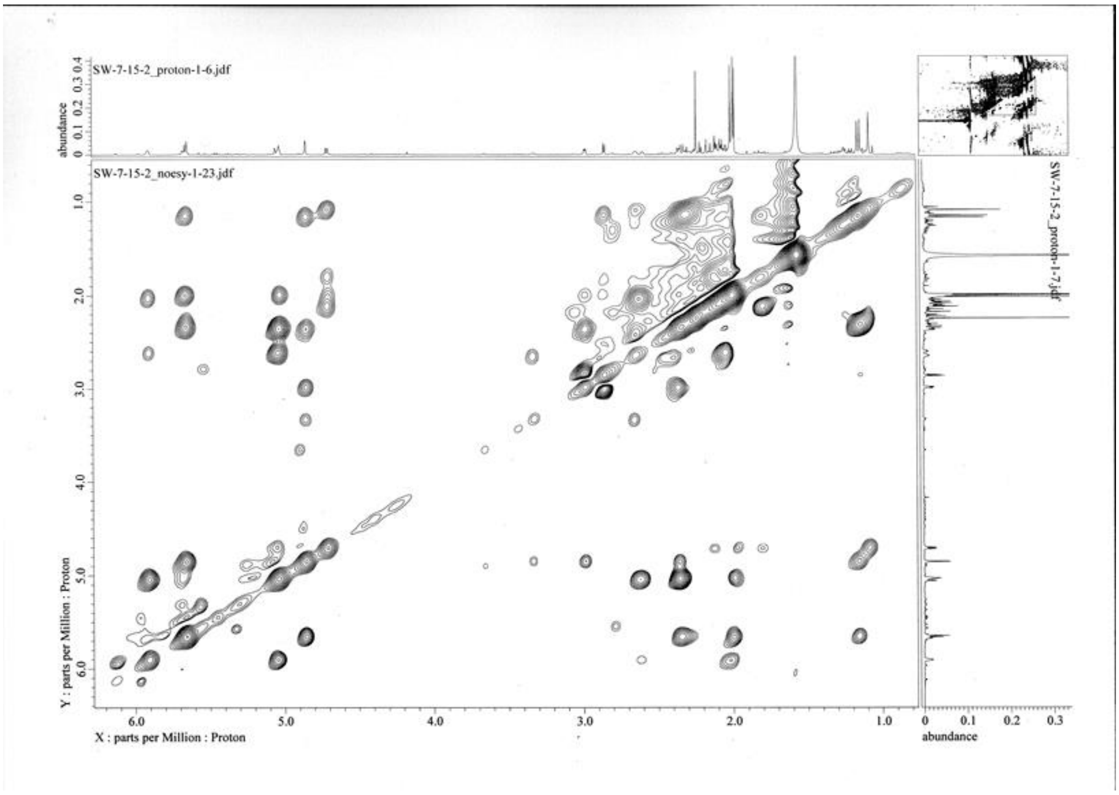

S8. NOESY spectrum of compound **1** in $CDCl_3$

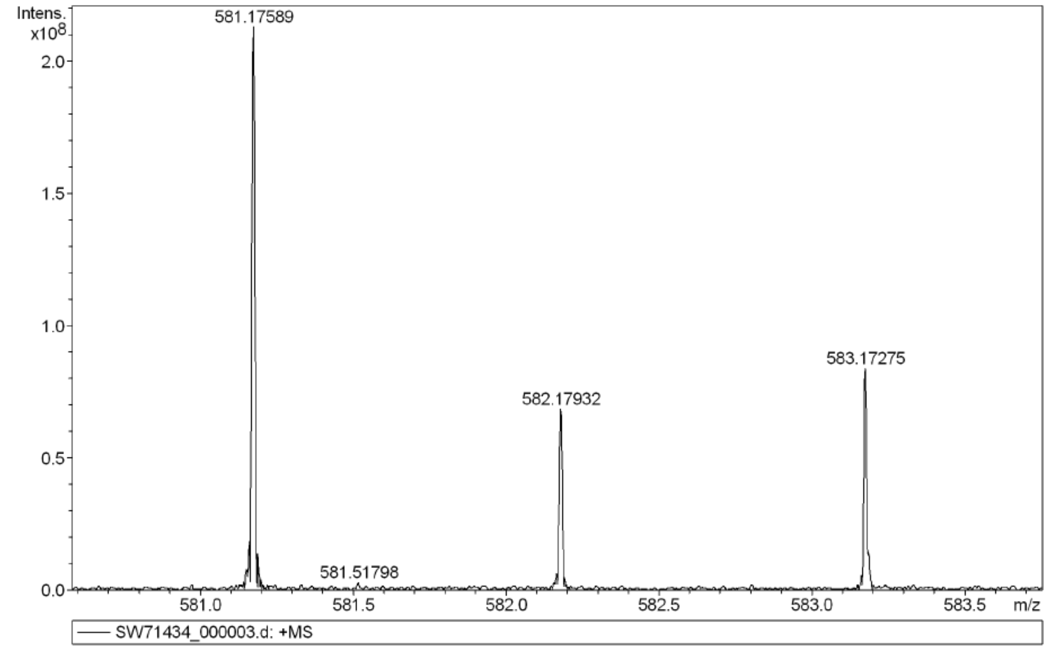

Meas. m/z	#	Formula	Score	m/z	err [mDa]	err [ppm]	mSigma	rdb	e⁻ Conf	N-Rule
581.17589	1	C 26 H 35 Cl Na O 11	100.00	581.17601	0.13	0.22	17.9	8.5	even	ok

S9. HRESIMS spectrum of compound **2**

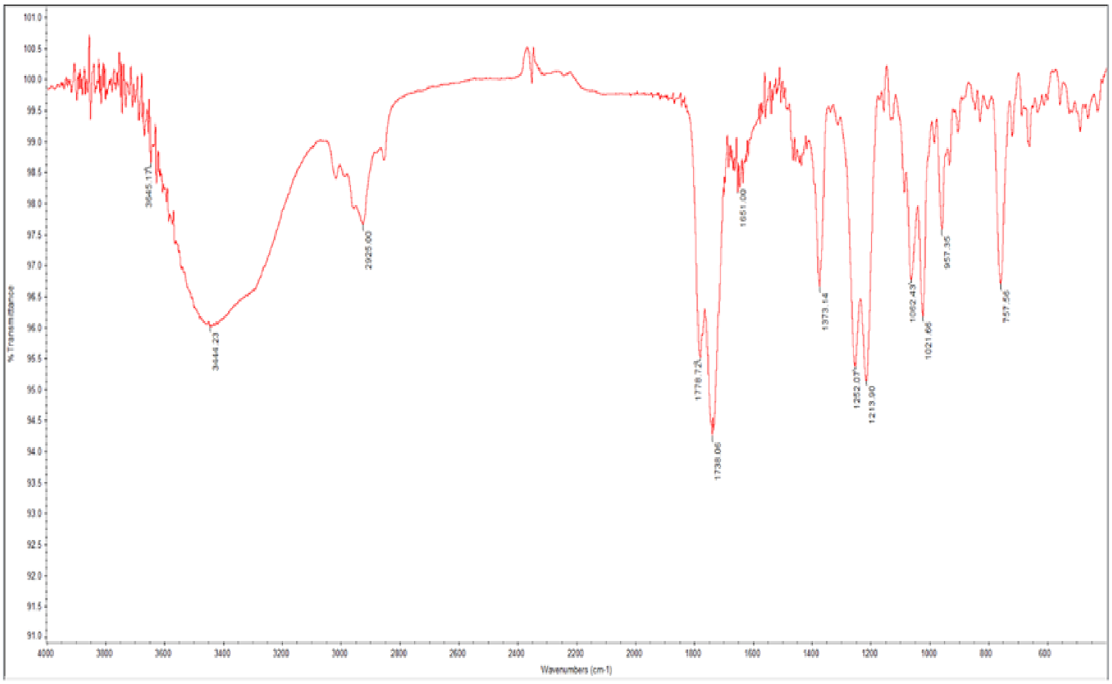

S10. IR spectrum of compound **2**

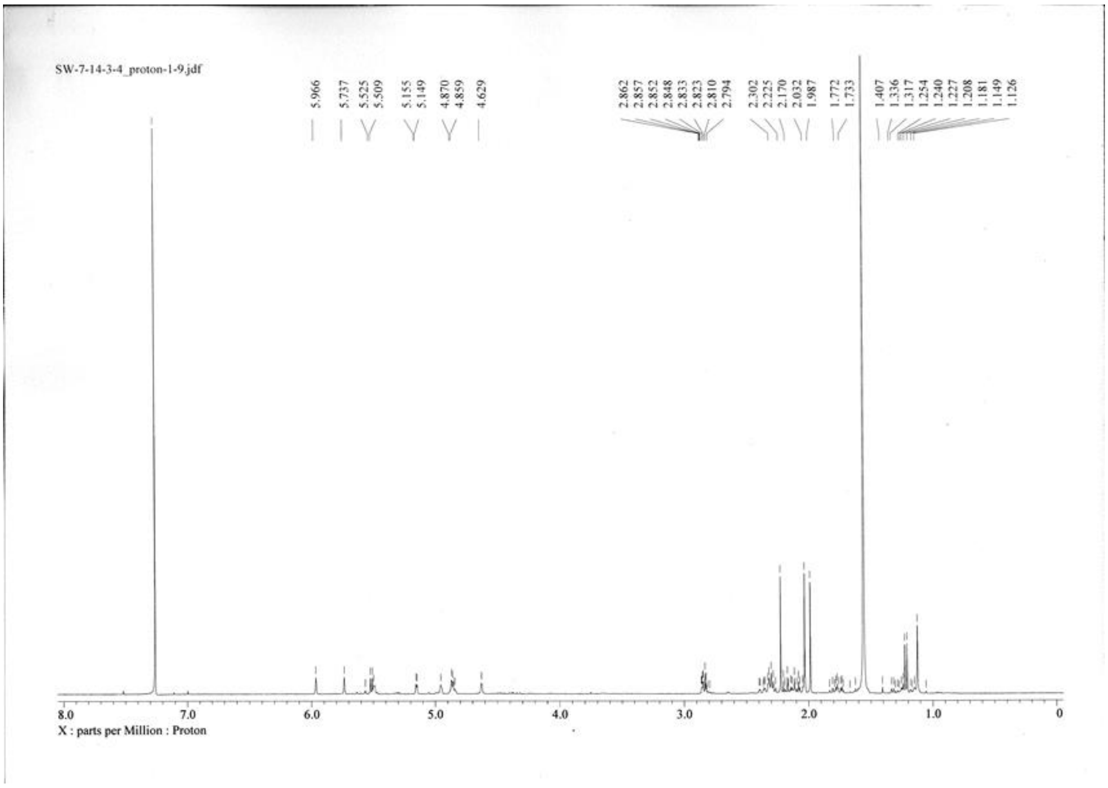

S11. ^{1}H NMR spectrum (400 MHz) of compound **2** in $CDCl_3$

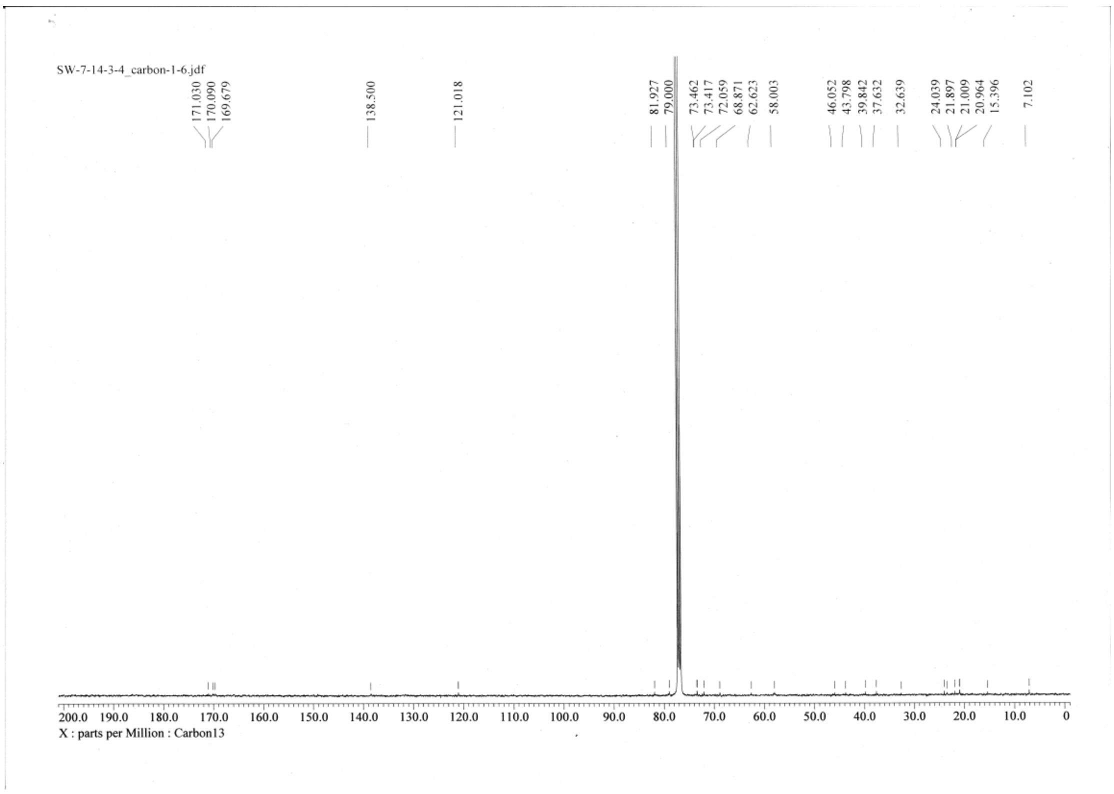

S12. ^{13}C NMR spectrum (100 MHz) of compound **2** in $CDCl_3$

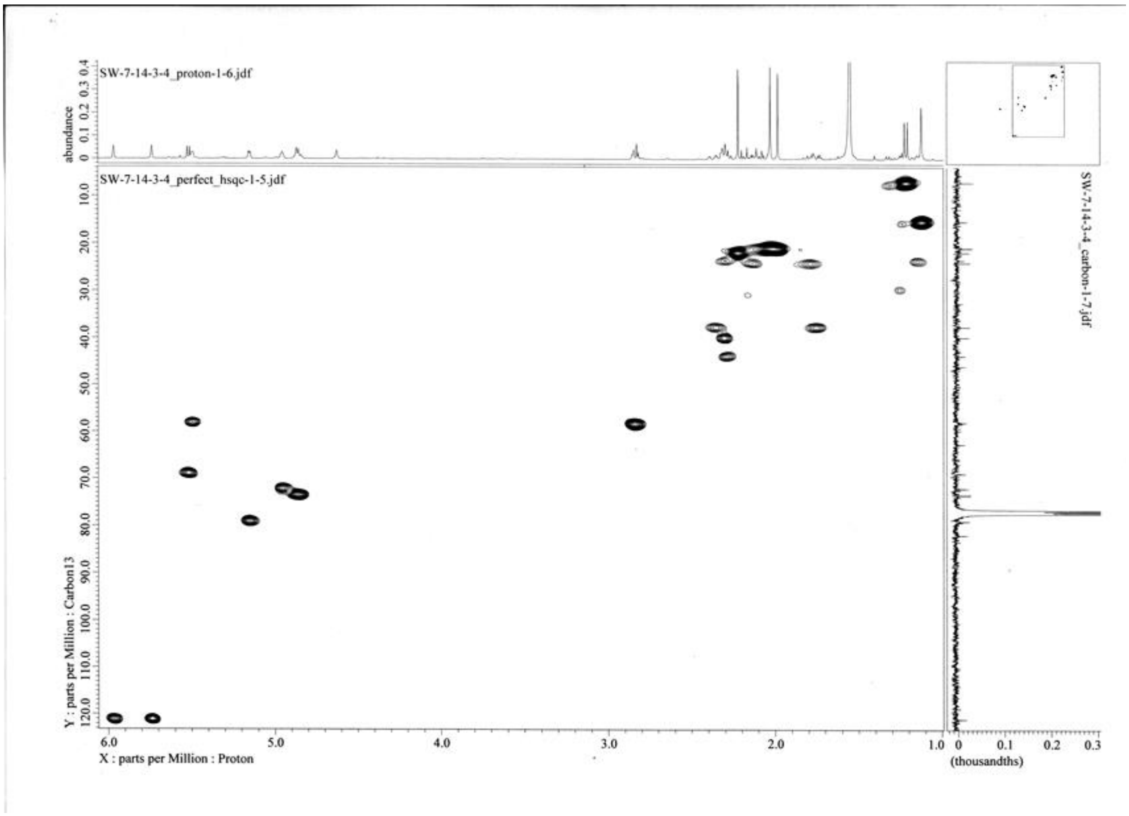

S13. HSQC spectrum of compound **2** in $CDCl_3$

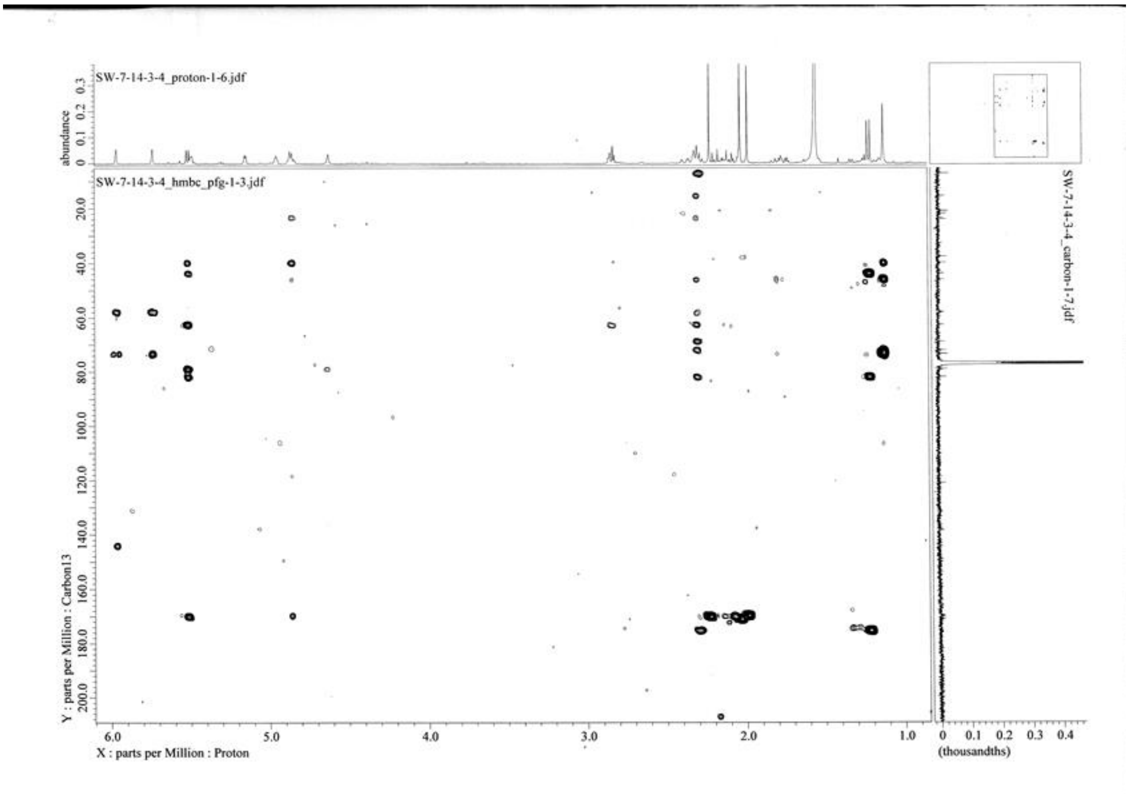

S14. HMBC spectrum of compound **2** in $CDCl_3$

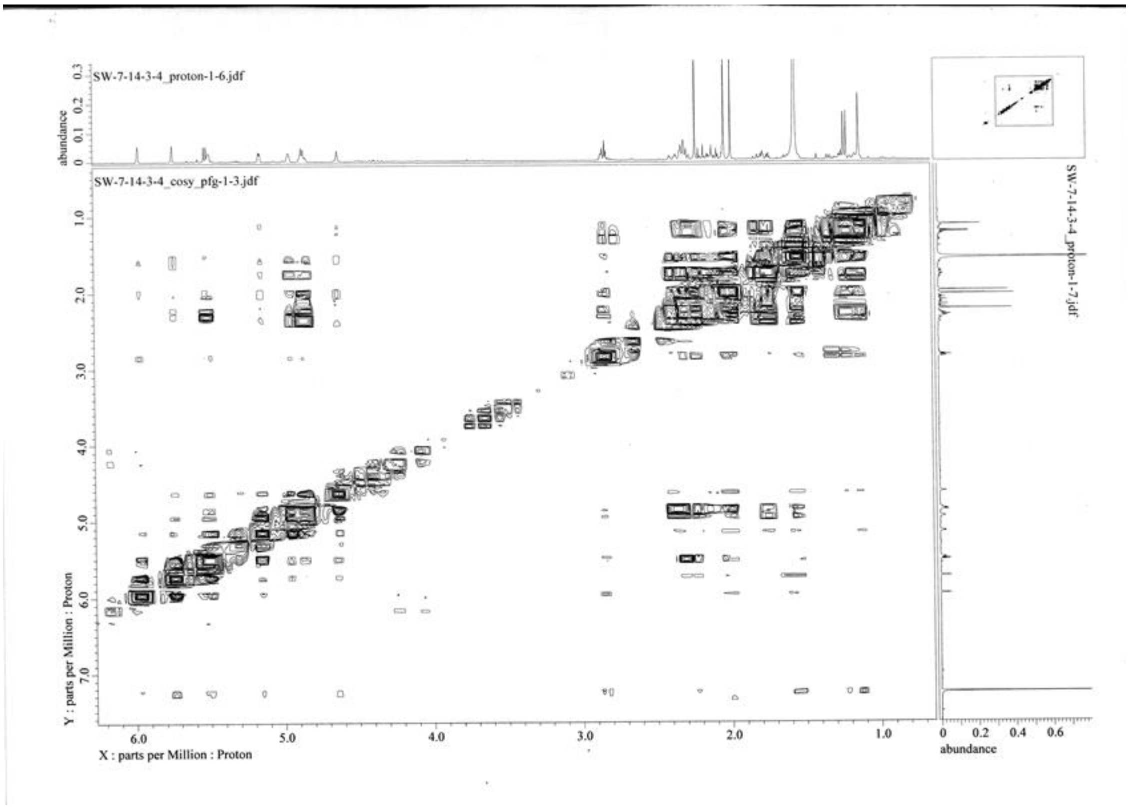

S15. ^{1}H-^{1}H COSY spectrum of compound **2** in $CDCl_3$

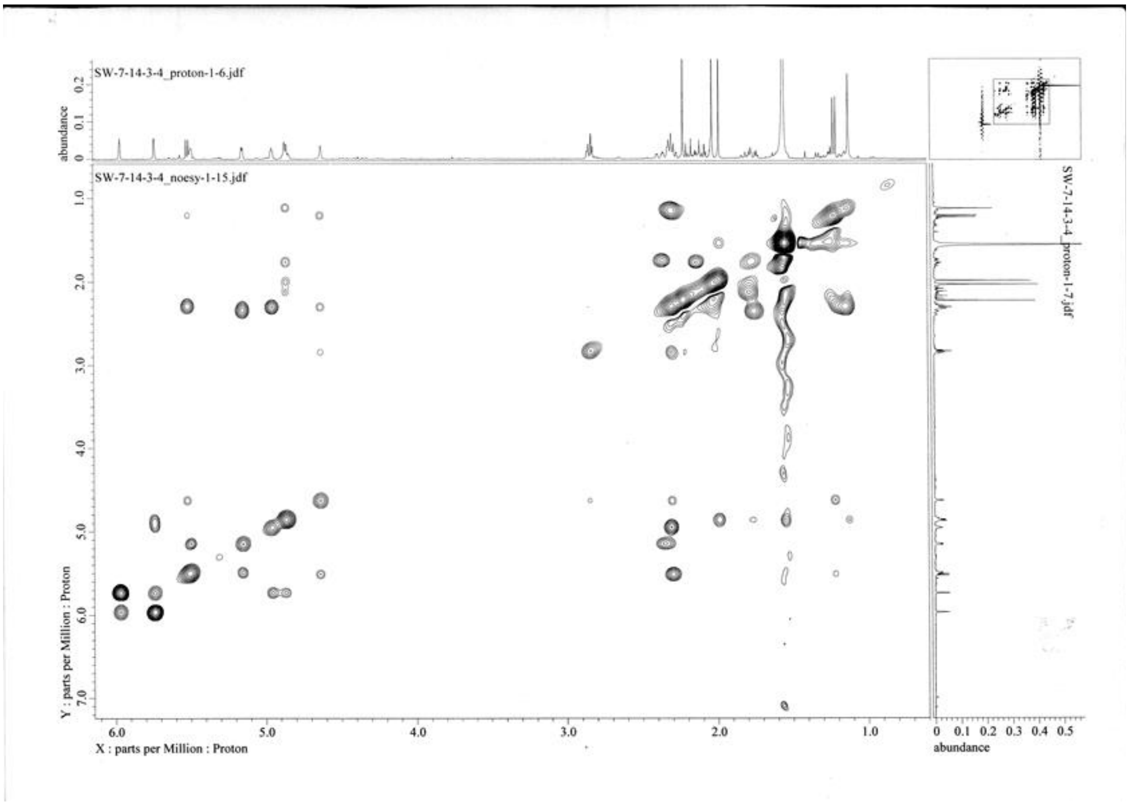

S16. NOESY spectrum of compound **2** in $CDCl_3$

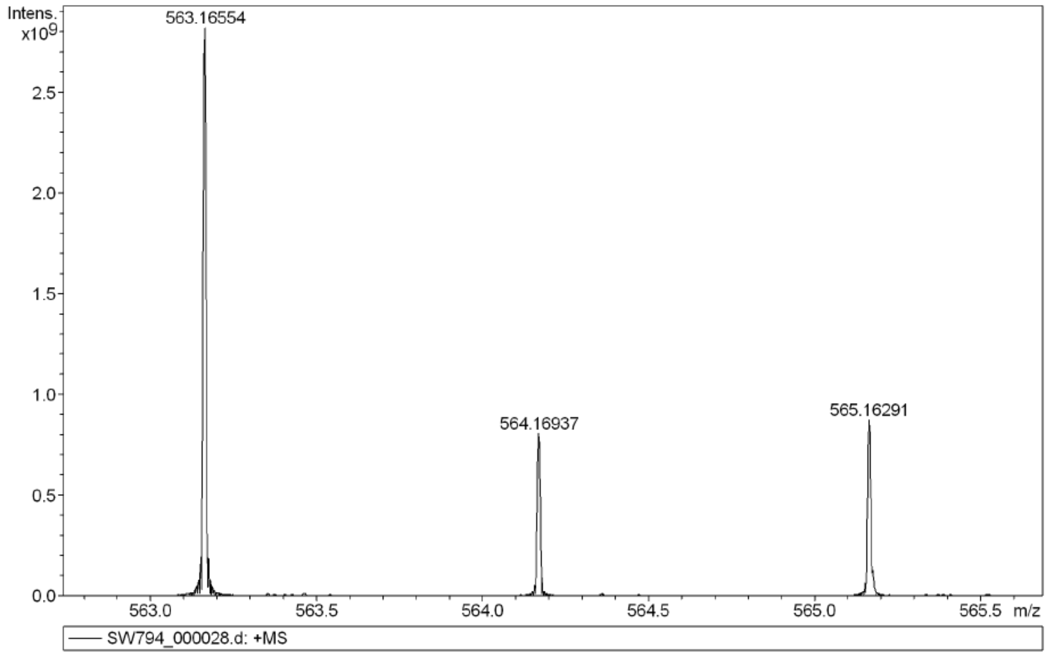

Meas. m/z	#	Formula	Score	m/z	err [mDa]	err [ppm]	mSigma	rdb	e⁻ Conf	N-Rule
563.16554	1	C 26 H 33 Cl Na O 10	100.00	563.16545	-0.10	-0.17	32.5	9.5	even	ok

S17. HRESIMS spectrum of compound **3**

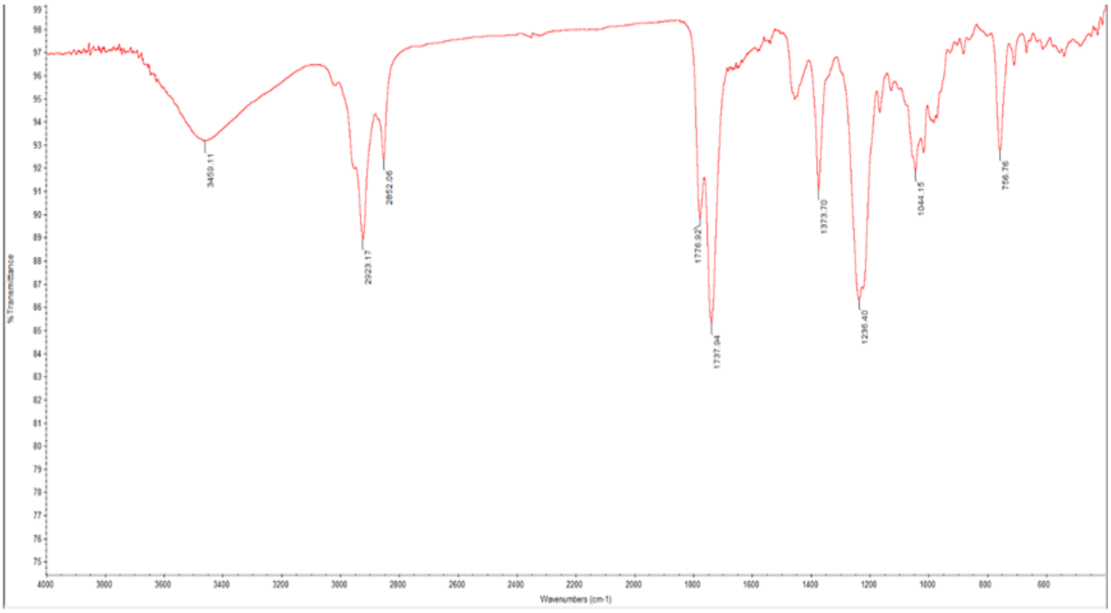

S18. IR spectrum of compound **3**

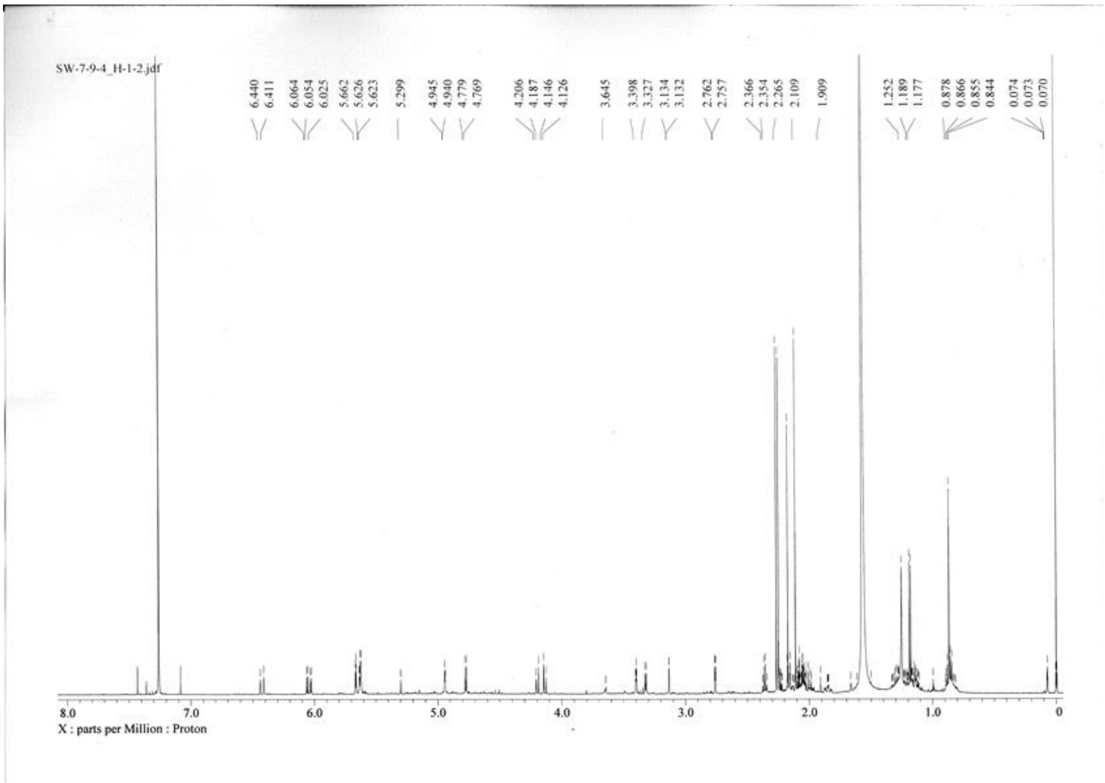

S19. ^{1}H NMR spectrum (600 MHz) of compound **3** in $CDCl_3$

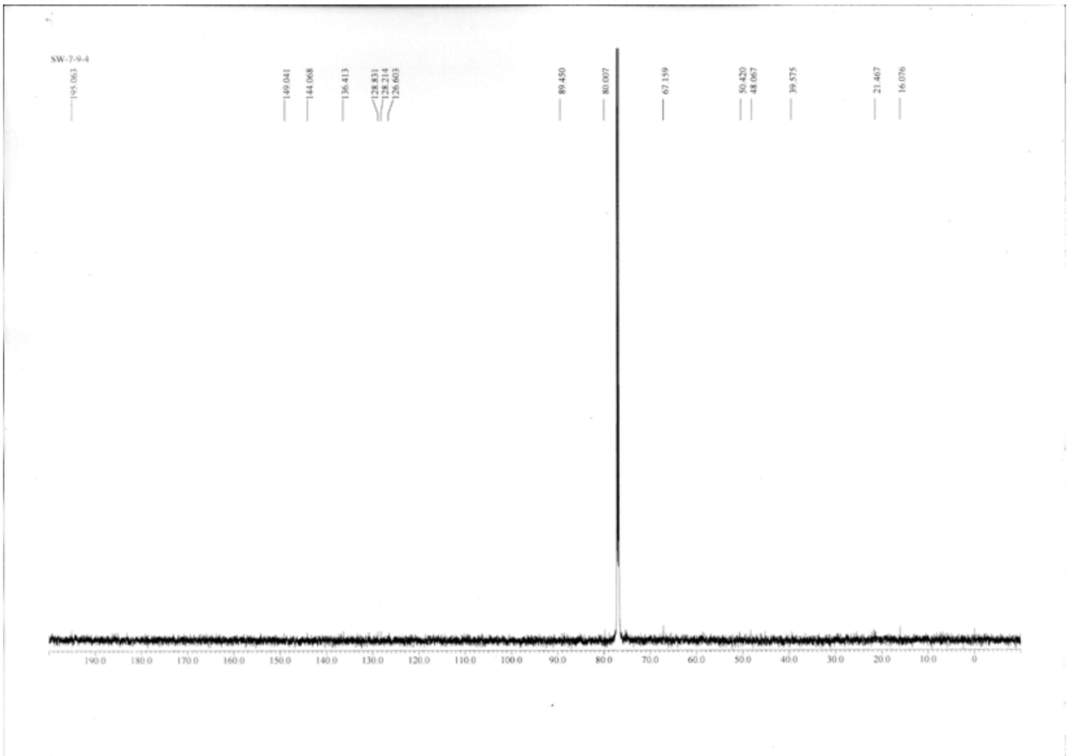

S20. ^{13}C NMR spectrum (150 MHz) of compound **3** in $CDCl_3$

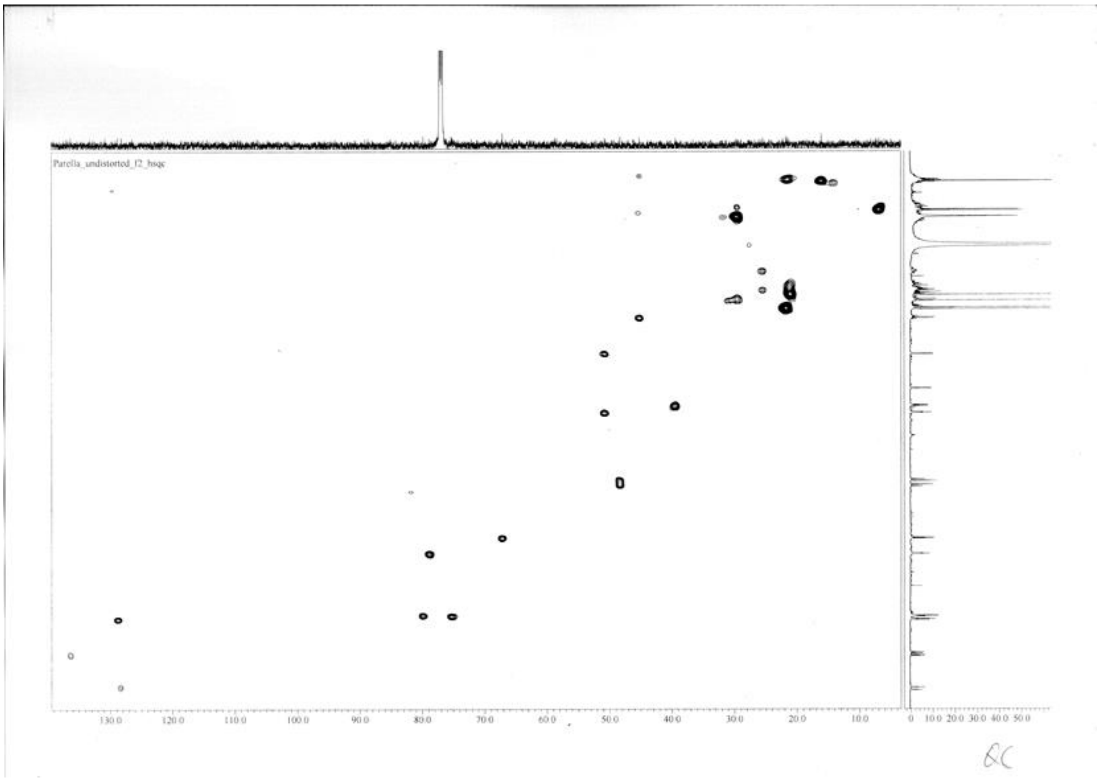

S21. HSQC spectrum of compound **3** in $CDCl_3$

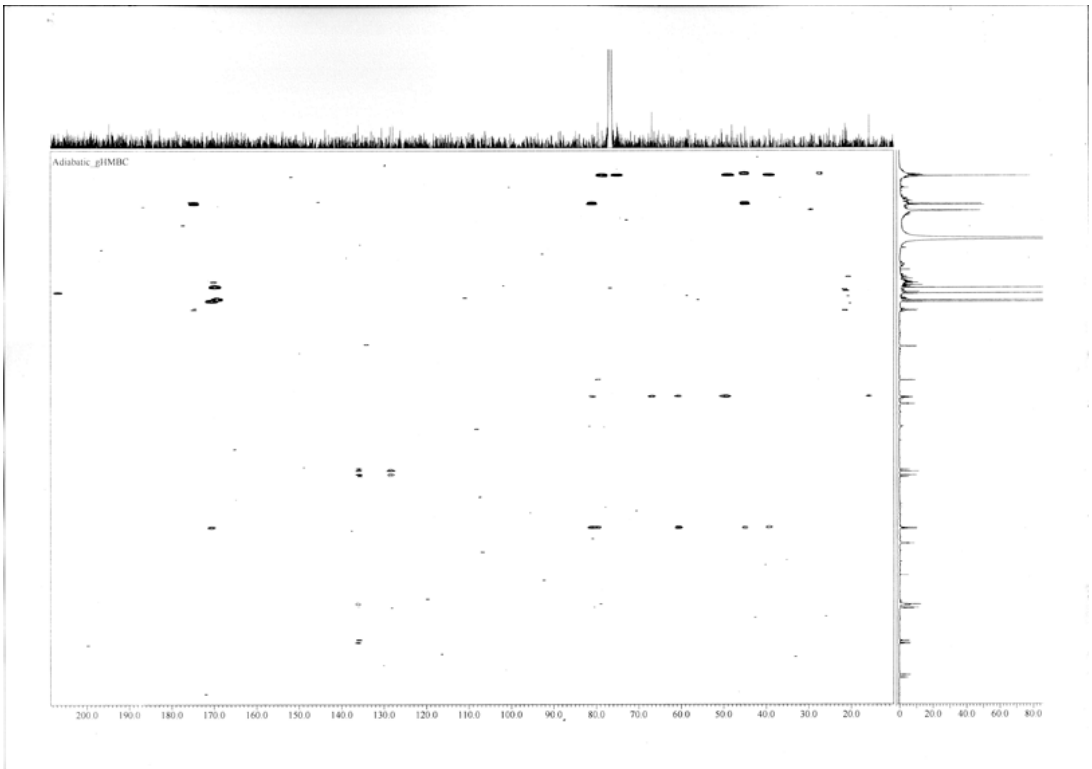

S22. HMBC spectrum of compound **3** in $CDCl_3$

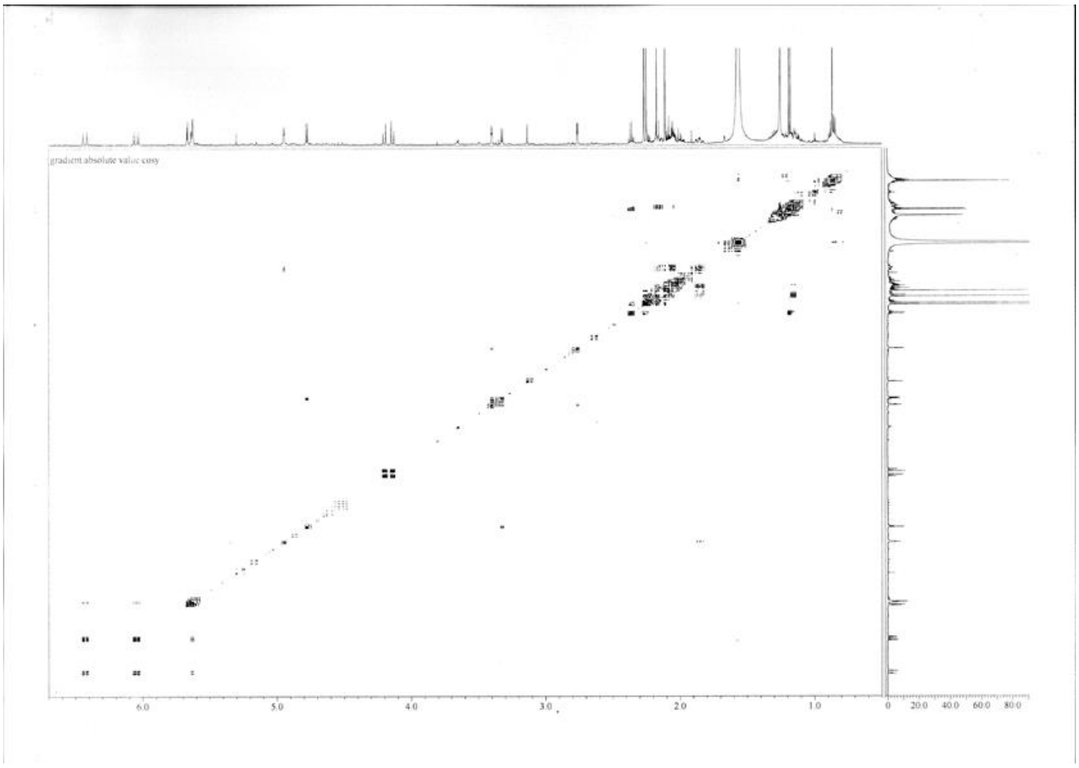

S23. ^{1}H-^{1}H COSY spectrum of compound **3** in $CDCl_3$

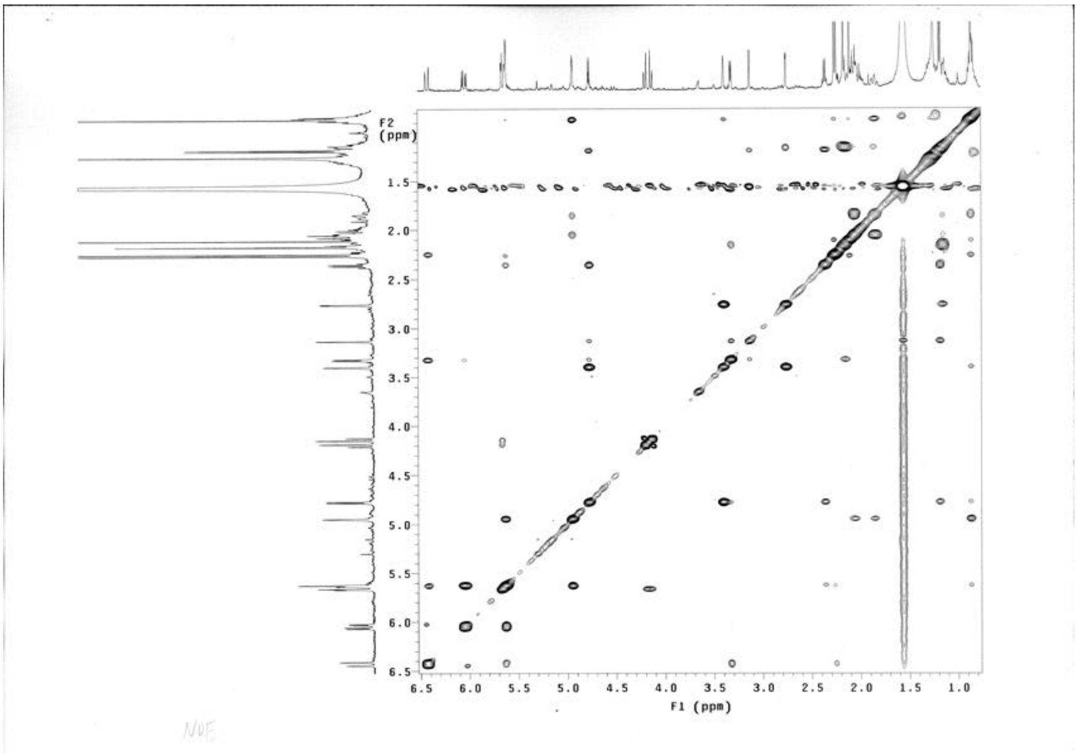

S24. NOESY spectrum of compound **3** in $CDCl_3$

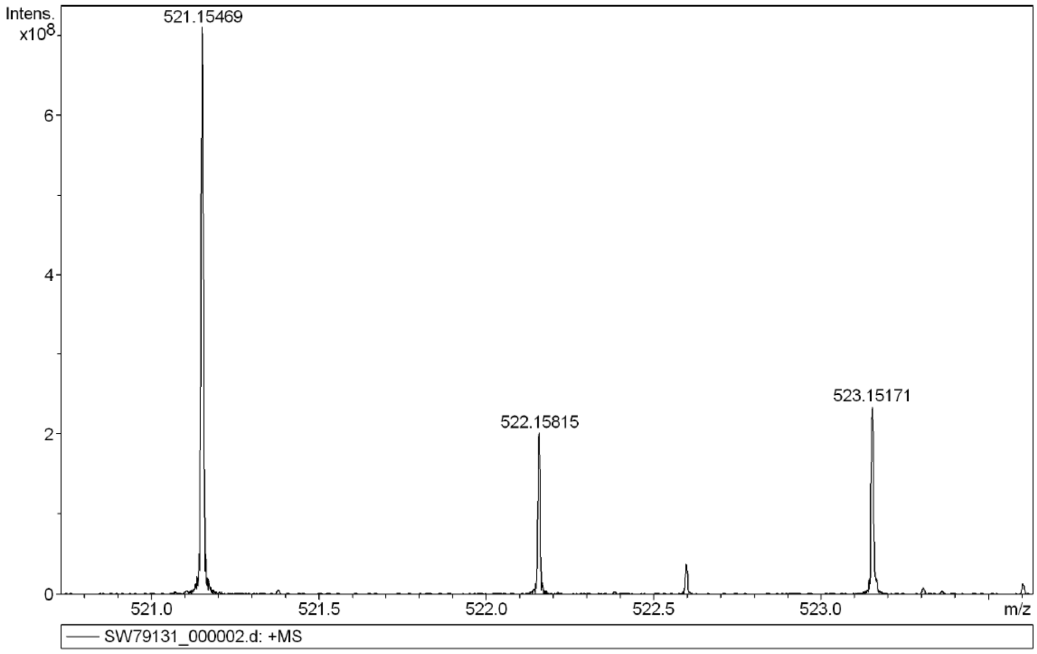

Meas. m/z	#	Formula	Score	m/z	err [mDa]	err [ppm]	mSigma	rdb	e⁻ Conf	N-Rule
521.15469	1	C 24 H 31 Cl Na O 9	100.00	521.15488	0.19	0.36	21.4	8.5	even	ok

S25. HRESIMS spectrum of compound **4**

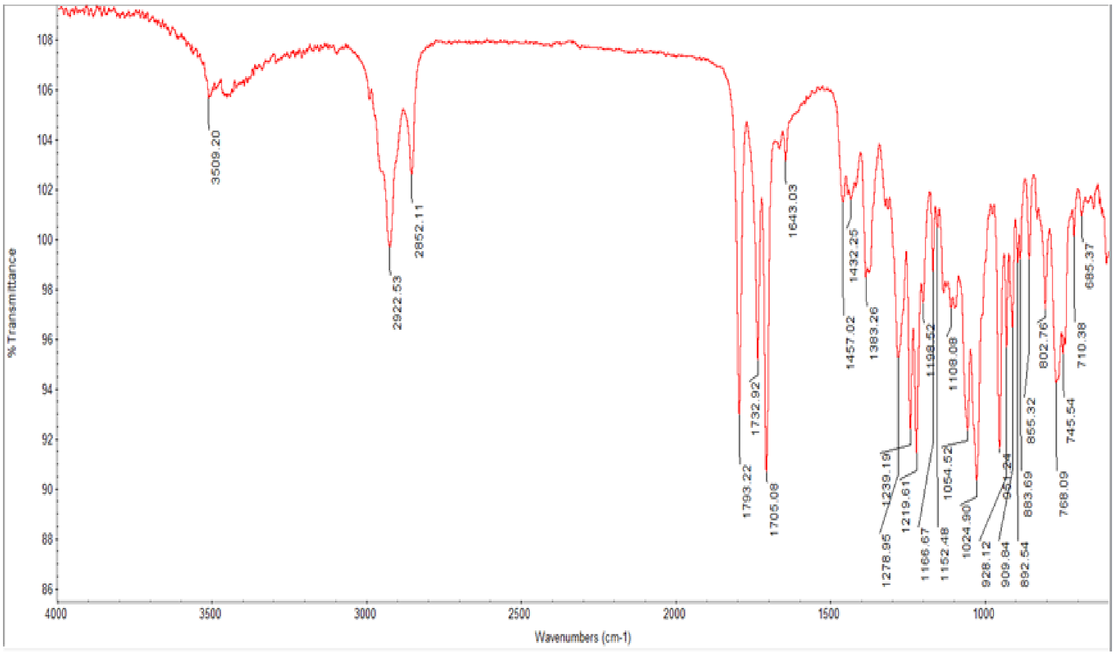

S26. IR spectrum of compound **4**

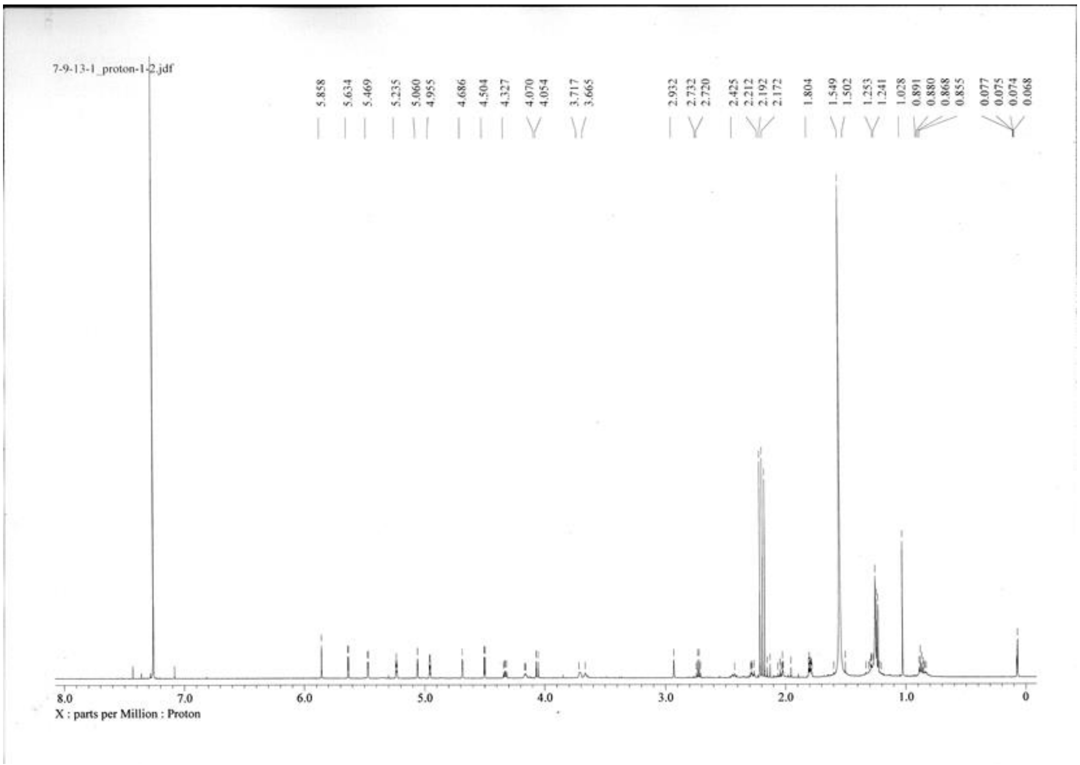

S27. ^{1}H NMR spectrum (600 MHz) of compound **4** in $CDCl_3$

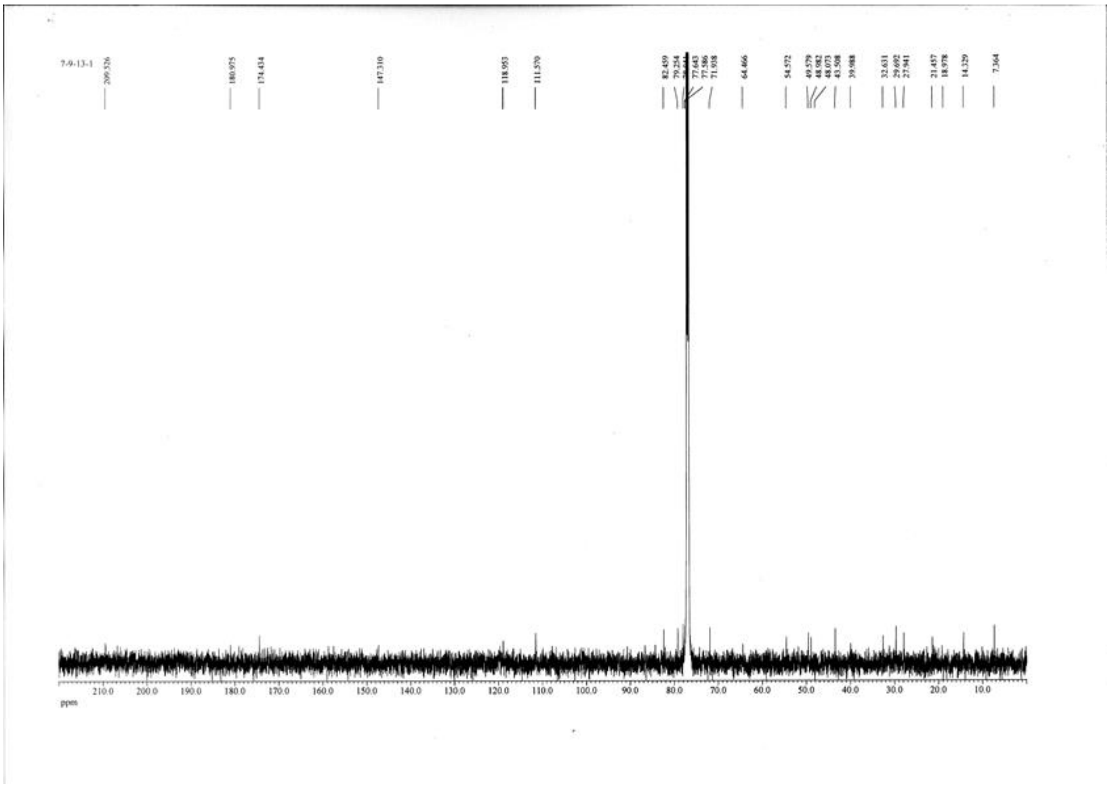

S28. ^{13}C NMR spectrum (150 MHz) of compound **4** in $CDCl_3$

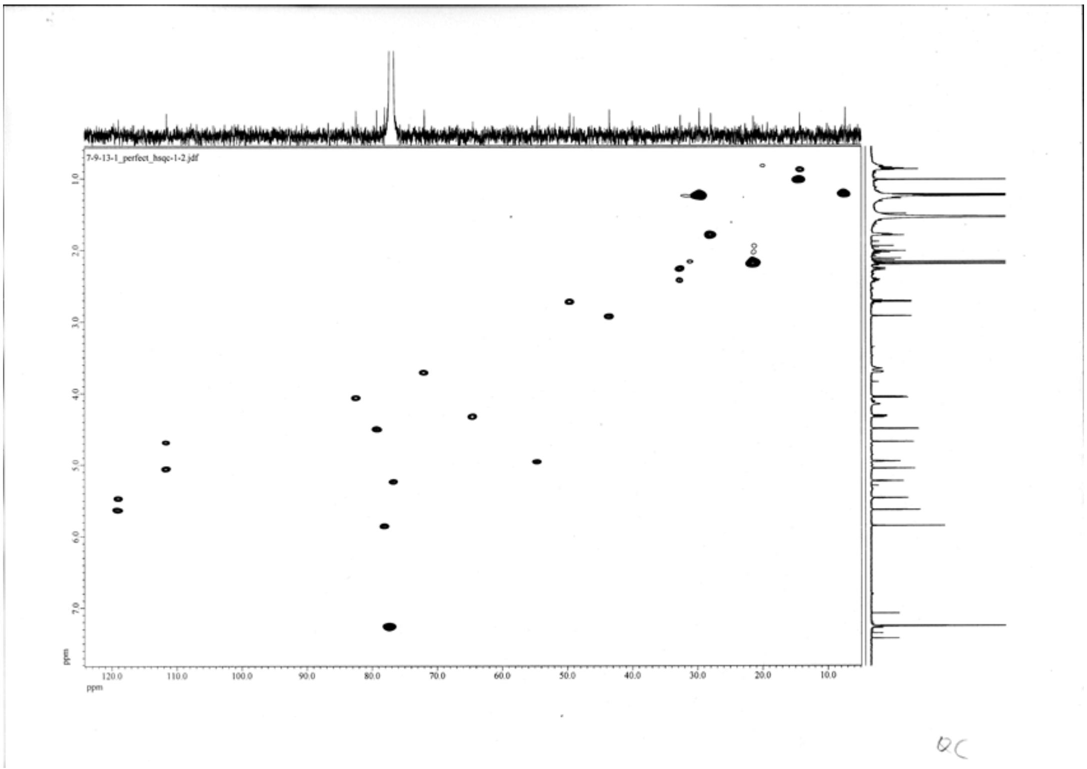

S29. HSQC spectrum of compound **4** in $CDCl_3$

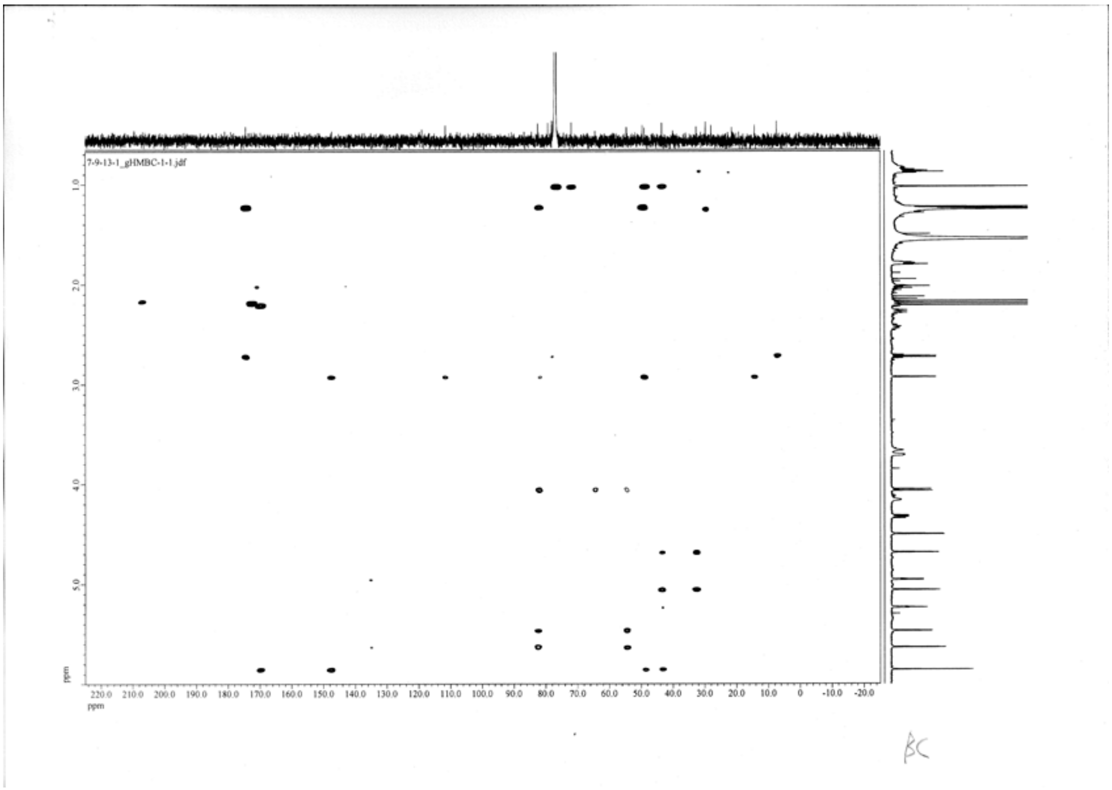

S30. HMBC spectrum of compound **4** in $CDCl_3$

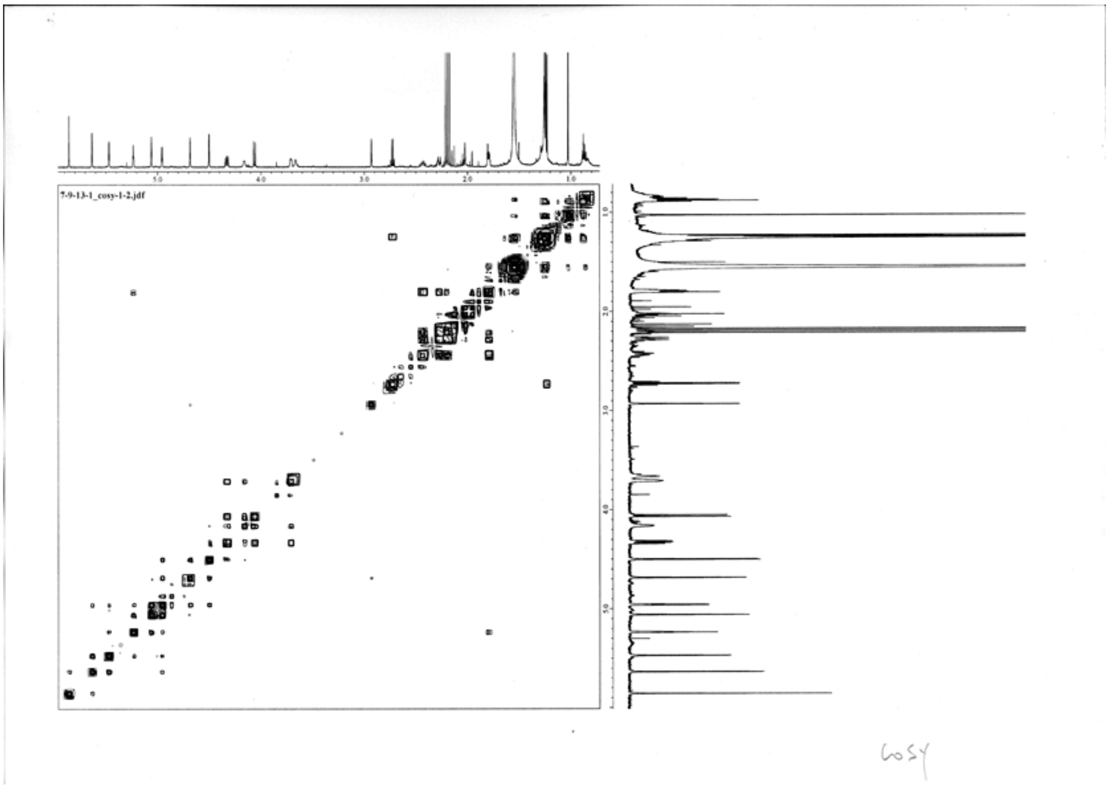

S31. ^1H-^1H COSY spectrum of compound **4** in $CDCl_3$

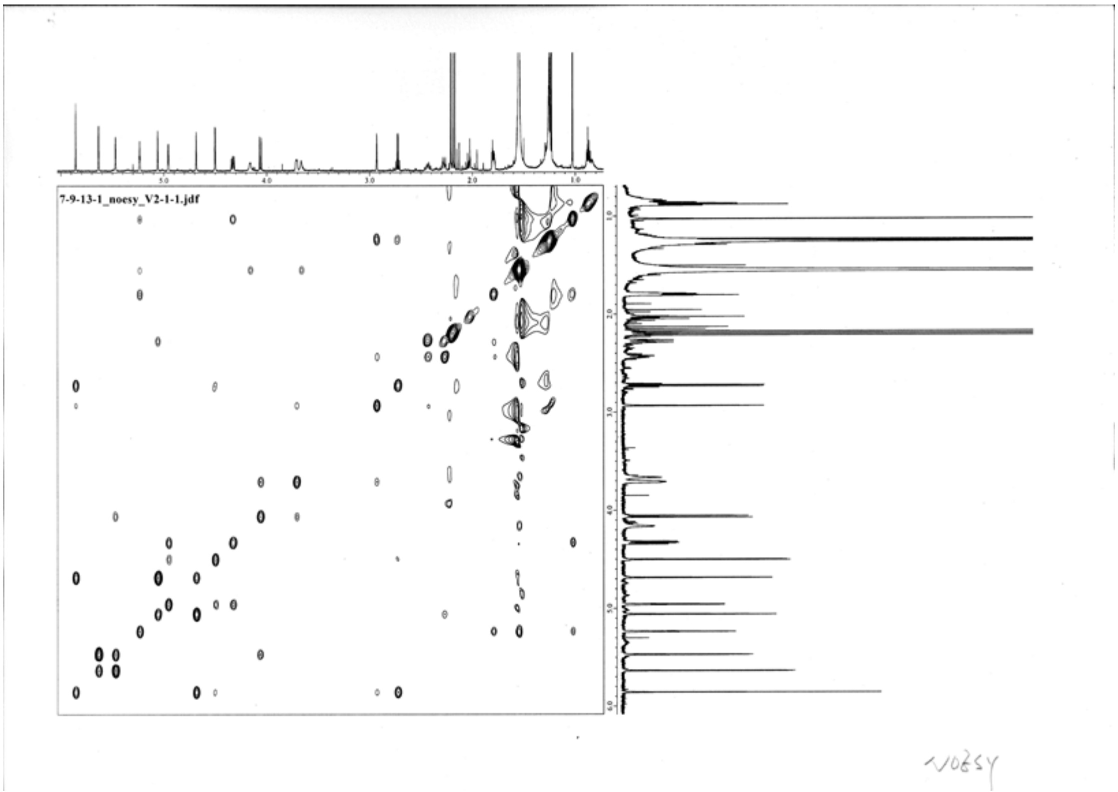

S32. NOESY spectrum of compound **4** in $CDCl_3$
